# Arabidopsis AGB1 participates in salinity response through bZIP17-mediated unfolded protein response

**DOI:** 10.1186/s12870-024-05296-x

**Published:** 2024-06-21

**Authors:** Yueh Cho

**Affiliations:** Institute of Plant and Microbial Biology, Academia Sinica, Taipei, 115201 Taiwan

**Keywords:** Heterotrimeric G protein, AGB1, Salinity, bZIP17, Unfolded protein response (UPR)

## Abstract

**Background:**

Plant heterotrimeric G proteins respond to various environmental stresses, including high salinity. It is known that Gβ subunit AGB1 functions in maintaining local and systemic Na^+^/K^+^ homeostasis to accommodate ionic toxicity under salt stress. However, whether AGB1 contributes to regulating gene expression for seedling’s survival under high salinity remains unclear.

**Results:**

We showed that AGB1-Venus localized to nuclei when facing excessive salt, and the induction of a set of bZIP17-dependent salt stress-responsive genes was reduced in the *agb1* mutant. We confirmed both genetic and physical interactions of AGB1 and bZIP17 in plant salinity response by comparing salt responses in the single and double mutants of *agb1* and *bzip17* and by BiFC assay, respectively. In addition, we show that AGB1 depletion decreases nuclei-localization of transgenic mRFP-bZIP17 under salt stress, as shown in *s1p s2p* double mutant in the Agrobacteria-mediated transient mRFP-bZIP17 expression in young seedlings.

**Conclusions:**

Our results indicate that AGB1 functions in S1P and/or S2P-mediated proteolytic processing of bZIP17 under salt stress to regulate the induction of salinity-responsive gene expression.

**Supplementary Information:**

The online version contains supplementary material available at 10.1186/s12870-024-05296-x.

## Background

Like every one of us, plants are facing an ever-changing environment day by day. Adjusting the armory to cope with divergent stresses is essential to equip seedlings with proper transcriptome for their fitness [1]. Integrating external stimuli into plant cells depends on hormones and heterotrimeric G proteins to deliver messages and initiate proper cellular responses for better survival [2]. The heterotrimeric guanine nucleotide-binding protein (G protein), including G⍺, Gβ, and Gγ subunits, serves as a signal mediator coupling with the plasma membrane-spanning G-protein-coupled receptors (GPCR) and effectors [3]. The human genome contains 23 G⍺ genes, 5 Gβ genes and 11 Gγ genes [4]. By contrast, Arabidopsis genome contains one canonical G PROTEIN ALPHA SUBUNIT 1 (GPA1) [5] and three non-canonical G⍺ subunits EXTRA-LARGE G-PROTEIN 1 (XLG1) [6], XLG2 and XLG3 [4], one Gβ subunit GTP BINDING PROTEIN BETA 1 (AGB1) [7] and three Gγ subunits G-PROTEIN GAMMA-SUBUNIT 1 (AGG1) [8], AGG2 [9], AGG3 [10]. Previous studies have shown that heterotrimeric G protein subunits play vital roles in responses to developmental cues and environmental stresses, including salt stress [11].

Soil salinity is one of the major threats to food security by seriously attenuating plant growth and decreasing crop yield [12]. Excessive salt in the soil causes numerous negative effects on different plant developmental stages, including germination, vegetative growth, and flowering [13]. These negative effects damage plant cells due to ion toxicity and increasing osmotic stress [14, 15]. Accordingly, plants have employed various mechanisms for survival in harsh salinity environments. Three signaling pathways constitute the major transduction during salt stress: calcium-dependent signaling pathway that (1) triggers the activation of stress-responsive genes as dehydration-responsive or late embryogenesis abundant (LEA) proteins [16] and (2) salt overly sensitive (SOS) pathway for regulation of ion homeostasis [17], and (3) osmotic stress signaling pathway involving ABA-dependent induction of downstream salt responsive genes through activation of a group of transcription factors like bZIP, NAC, MYB and ABRE families [18]. However, our understanding of the whole network of pathways regulating salinity response is far from complete.

Recent research based on studies of Arabidopsis AGB1 has suggested a functional link between plant G protein signaling and regulation of the salt stress response. A knockout mutant of *AGB1* exhibited more sensitivity to high salinity than wild-type plants [10, 11, 19–21]. Under excessive salt, the *agb1* mutant accumulates more Na^+^, translocate more Na^+^ from root to shoot, and has a high transpiration rate with larger stomatal apertures [22, 23]; AGB1 is also coupled with AGG1 or AGG2 to regulate stomatal apertures and transpiration [24]. Meanwhile, a receptor-like kinase, FERONIA (FER), has been identified by directed interaction with AGB1. FER is required for cell wall integrity, Ca2^+^ induction, ROS production, and stomata movement under salinity conditions [25].

Most G protein signaling studies have focused on canonical effectors localized to the plasma membrane. Recent studies have shown that the G-protein β subunit functions in the nucleus [23, 26]. The Arabidopsis AGB1 is localized in the nucleus, where it interacts with B-BOX DOMAIN PROTEIN 21 (BBX21) for hypocotyl elongation [27], with BRI1-EMS-SUPPRESSOR 1 (BES1) for cell division [28], with PHYTOCHROME B (phyB)—PHYTOCHROME INTERACTING FACTOR 3 (PIF3) [29] or CRYPTOCHROME 1 (CRY1)—ELONGATED HYPOCOTYL 5 (HY5) for photomorphogenesis [30] and with MAP KINASE 6 (MPK6) for drought tolerance [31]. Nonetheless, gaining evidence showed that WD domain-containing proteins, including AGB1, directly interacted with bZIP transcription factors like HY5 and VIRE2-INTERACTING PROTEIN 1 (VIP1) to regulate gene expressions [30, 31].

The basic leucine zippers transcription (bZIP) family in Arabidopsis comprised 78 members and assorted into 13 groups [32]. bZIPs form as dimers to bind DNA sequences, and heterodimerization results in appreciable regulatory flexibility [33]. Among these bZIP transcription factors, three members of group B (bZIP17, bZIP28, bZIP49) perform as important regulators of the evolutionally conserved ER stress response [34], the intrafamily dimerization of group B has been confirmed in yeast cells [35]. In particular, under adverse environmental conditions, including high salinity, bZIP17 is reported to relocate from ER to nucleus processing by through regulated intramembrane proteolysis [36]. Salt-responsive genes are reported to express in a bZIP17-dependent manner [36, 37]. In this study, we observed the nuclei localization and the contributions of AGB1 to induce the expression of bZIP17-mediated salinity-responsive genes. The spliced bZIP17 localization toward nuclei through SITE-1 PROTEASE (S1P) / SITE-2 PROTEASE (S2P)-mediated proteolysis was reduced without AGB1. The arrangement of AGB1 pools among different subcellular compartments to involve proper programs to respond to high salinity is crucial for young seedling viability.

## Results

### Functional complementation of salinity hypersensitivity in *agb1* mutant

**Fig. 1:**
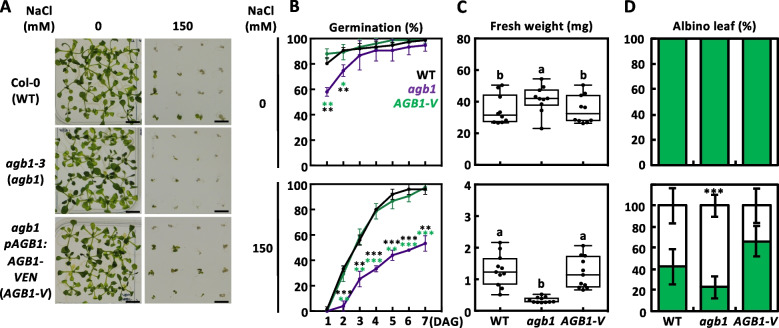
Functional complementation of *agb1* hypersensitivity to salt stress. **A** Salinity tolerance assay, **B** germination rates, **C** fresh weight, and **D** percentage of the albino leaf of *agb1-3* (*agb1*) mutant and *agb1 pAGB1:AGB1-VEN* (*AGB1-V*) transgenic plant seeds compared with the corresponding Col-0 (WT) seeds. Representative images (**A**) of 14-day-old WT, *agb1* mutant, and *AGB1-V* transgenic plant were grown on ½ MS containing NaCl with indicated concentration to induce salt stress. **B** Each value represents the means ± SD of the germination percentage (with 25 seeds) for six independent experiments. **C** Representative plots for fresh weight of 14-day-old seedlings grown on 0 (up) or 150 mM NaCl (down) were measured individually with six biological experiments (*n* = 11) and shown as means ± SD. Data with different letters represent significant differences [one-way ANOVA at *P* < 0.05]. **D** The *albino* leaves were counted and displayed as means ± SD. [Student t-test, ***, *P* < 0.001].

In this study, the *AGB1* transcript was significantly increased under high salt stress after 4 h, as described previously (Fig. S3A; [19]). The *agb1-3* mutant is a null mutant of *AGB1* (as *agb1*, Fig. S1A, S1B, S2A, S2B [38]). It shows hypersensitive responses, including poor germination, growth defects, and *albino* leaves when grown under salt stress (Fig. 1). We produced the genetic complementation lines harboring genomic sequence of *AGB1* as previously described (*agb1 pAGB1:AGB1,* as *AGB1*; [38]) with C-terminally fused the triple Venus fluorescent protein in the *agb1* mutant (*agb1 pAGB1:AGB1-Venus*, as *AGB1-V*; Fig. S1A, S1B and S1C). To test whether *AGB1-V* transgene is functional in vivo, we observed shoot, primary root, and lateral roots. The *AGB1*, *AGB1-V* transgenic plants rescued the developmental defects in *agb1* mutant (Fig. 1, S1D, S1E, and S1F). We then performed the sequential salt stress tolerance assay on wild-type (WT), *agb1* mutant, and *AGB1-V* plants for 14 days after seeds sowing on ½ MS medium (Fig. 1 and S3). The leaf bleaching phenotype and growth retardation in *agb1* mutant were fully rescued in the *AGB1-V* complementation lines (Fig. 1A, 1C, 1D, S3B, S3C), which were either counted for the *albino* rate (Fig. 1D) or classified into three groups according to the seedling size and chlorotic phenotypes as Green, Mix (contained at least one white leaf) and White (Fig. S3B, S3C). In the mock treatment (0 mM NaCl), all WT, *agb1,* and *AGB1-V* seedlings grew normally and showed green for their aerial part. In contrast, in the salt stress treatment (150 mM NaCl), more *agb1* seedlings were categorized significantly into the white group due to the chlorotic phenotype compared to the WT and *AGB1-V* seedlings (Fig. 1D, S3B, S3C). Since no statistical difference was found between WT and *AGB1-V* seedlings, we suggested that the *AGB1-V* was functional and complemented the *agb1* mutant in vivo (Fig. 1, S1, S2C, S2D and S3)*.*

### Subcellular localization of AGB1 at the nuclei in response to salt stress

**Fig. 2:**
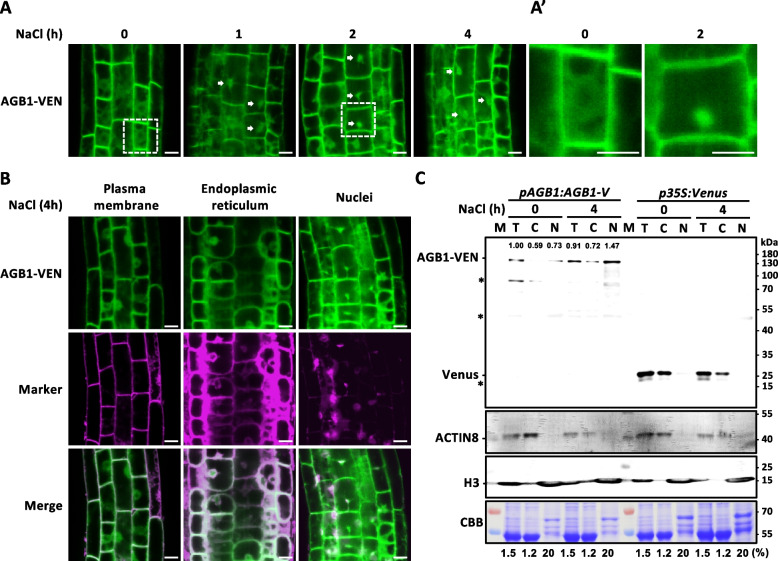
Nuclei localization of AGB1 in response to high salinity. **A** Representative images of ProAGB1:AGB1-VENUS (AGB1-VEN) in a stable transgenic complementation plant in the absence (½ MS medium) or presence of 150 mM NaCl as indicated time for salt stress induction. White arrows indicated the nuclei localization of AGB1. The regions of the root epidermis were marked by dash open boxes and zoomed to show in the right (**A’**). **B** Subcellular localization of AGB1 in root of 7-day-old *AGB1-V* plant. Fluorescence of AGB1-VEN (Green) and staining of the plasma membrane by FM4-64 (left), endoplasmic reticulum (ER) by ER-tracker (middle), and nuclei by DAPI (right) in treatment of 150 mM NaCl for 0 or 4 h. Co-localization signals of AGB1-VEN and staining dye were shown in the merge images as white colors in the bottom panel. Scale bars equal to 10 µm. **C** Immunoblot analysis of AGB1-VEN protein among different sub-cellular fractions. Nuclei isolation of 7-day-old *AGB1-V* seedlings with 0 or 4 h of 150 mM NaCl treatment, cytosol (C), or nuclei (N) fractions were separated after centrifugation. T: total input. The percentages of each fraction for sample loading were indicated in the bottom per lane. Immunoblots of AGB1-VEN (121 kDa) were detected by anti-GFP antibody, anti-ACTIN8 antibody was used as cytosol marker, and anti-histone H3 antibody (H3: 17 kDa) was used as nuclei marker. The asterisks indicated the non-specific bands. CBB staining as the loading control. The relative ratio of AGB1-VEN was normalized to CBB intensity

AGB1 is known as a molecular switch through protein-protein interactions to regulate transcriptional programs for plant development and abiotic stress responses, like inhibiting BBX21 activation [27] or binding with PIF3 in the nucleus to promote hypocotyl elongation [29], dephosphorylating BES1 for its nuclei entry and downstream gene expression [28], which prompted us to investigate whether AGB1 localization into nuclei to involve transcriptional regulation under high salinity. The 7-day-old transgenic *AGB1-V* seedlings were used for confocal laser scanning microscopy to observe any changes in subcellular localization of AGB1-Venus (AGB1-VEN) that occurred during short-term NaCl treatment. The time series of salt treatment continuously tracked AGB1-VEN’s localization in the root epidermis (Fig. 2A). Most of AGB1-VEN showed network-like localization under normal conditions, which prompted us to consider the possible ER localization of AGB1 as a previous study described (*ProAGB1:CFP-AGB1* [39]; *Pro35S: AGB1-GFP* [26]). In contrast, many AGB1-Venus expressions showed nuclei-like patterns in the *AGB1-V* root under 1, 2, and 4 h of salt stress (Fig. 2A) as shown in enlarged images (Fig. 2A’). This nuclei localization of AGB1-VEN can be seen in other tissues like hypocotyl (middle) and also cotyledon (right) in a spatiotemporal manner (Fig. S6A and S6B); the dot-like are confirmed by DAPI staining to mark nuclei (4-h NaCl treatment, red in Fig. S6A). The nuclei-localized AGB1-VEN was rarely seen in the tissue with 0-h NaCl treatment, found in the tissue with 1- to 16-h NaCl treatment as a solid triangle indicated in Fig. S6A. To quantify the nuclei-localized AGB1-VEN, we grouped the ER-like and PM-like-localized AGB1-VEN cells (black group) and counted the ratio of the different localized AGB1-VEN in the tissue that has been examined (Fig. S6B). The nuclei-localized AGB1-VEN cells (white group) were significantly found in all NaCl-treated tissue with various lengths of time for salt stress induction (Fig. S6B).

We also stained 7-day-old *AGB1-V* seedlings with plasma membrane (PM) marker (FM4-64, Fig. 2B, Left) and ER marker (ER-tracker, Fig. 2B, middle), nuclei marker (DAPI, Fig. 2B, right). The AGB1-VEN signals were partially colocalized with organelle and nuclear markers in the presence of salt stress after 4 h. The FM4-64 patterns matched cell boundaries, the ER tracker marked the perinuclear ER network, and DAPI filled the central pot inside of the ER (Fig. 2). Next, we performed the fractionation assay to isolate nuclei from 7-day-old *AGB1-V* seedlings with 0- or 4-h NaCl treatment, and the immunoblots of ACTIN8 and Histone H3 served as cytosolic and nuclear marker, respectively. The AGB1-VEN proteins were quantified with CBB staining and *p35S: Venus* acted as a negative control for Venus in nuclei fraction. The proportion of AGB1-Venus increased around twofold in nuclei fraction under salt stress (0.73 to 1.47), but there was no obvious change for AGB1-VEN in total and cytosolic fractions. The above results indicate that AGB1-VEN is widely distributed to PM and ER under normal conditions, which is increasingly localized to nuclei and likely reduced ER localization of AGB1-VEN in response to salt stress (Fig. 2 and S6).

### Repression of transcription factor bZIP17-mediated salt stress response in *agb1* mutant

**Fig. 3:**
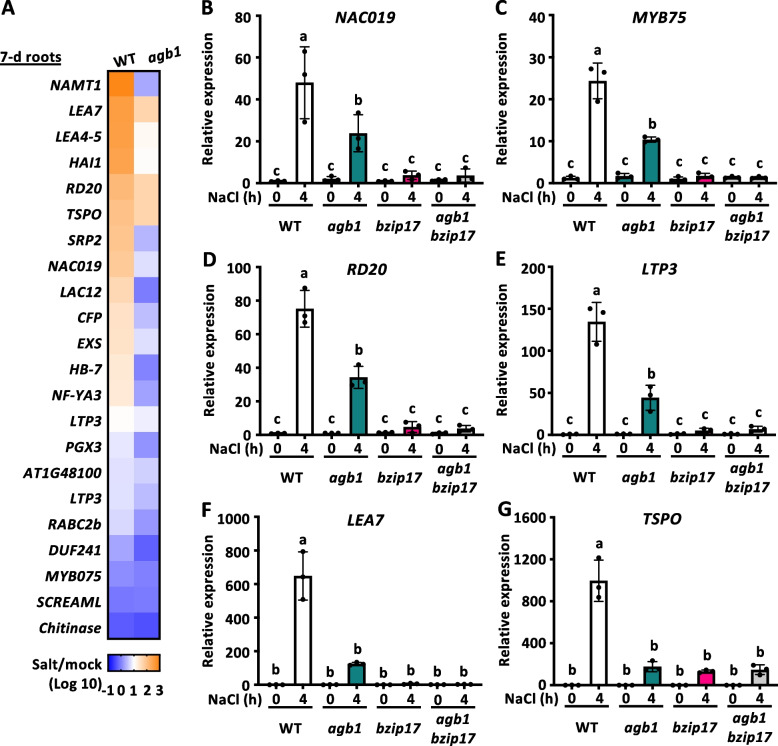
Salt stress induction of bZIP17-mediated salt stress response in *agb1* mutant. **A** Twenty-two bZIP17-mediated salt stress responsive genes were selected from Liu et al., [36] and their expressions under salt stress in 7-day-old WT or *agb1-3* (*agb1*) mutant were shown as heatmap. The roots were collected for microarray analysis after 0 (control) or 4 h (salt) of 150 mM NaCl treatment. The Log10 values indicated the means of the ratio of the representative transcripts under salt stress (4-h) comparing the mock condition in WT or *agb1* mutant from three independent replicates. **B** *NAC DOMAIN CONTAINING PROTEIN 19* (*NAC019*), **C** *MYB DOMAIN PROTEIN 75* (*MYB75*), **D** *RESPONSIVE TO DESICCATION 20* (*RD20*), **E** *LIPID TRANSFER PROTEIN3* (*LTP3*), **F** *LATE EMBRYOGENESIS ABUNDANT PROTEIN7* (*LEA7*), **G** *OUTER MEMBRANE TRYPTOPHAN-RICH SENSORY PROTEIN)-RELATED* (*TSPO*) were quantified. The expression of the WT sample at 0 h set to 1. Three technical replicates averaged data in the same run, and three biological replicates in separate runs were shown in mean ± SD. Data with different letters represent significant differences [one-way ANOVA at *P* < 0.05]

We wonder whether AGB1 played a role in transcriptional regulation in response to salt stress since gaining portions of AGB1-VEN to nuclei was observed in young seedlings; if so, what would be the corresponding gene sets to develop protection against excessive salt? We therefore performed microarray analyses using 7-day-old WT and *agb1* whole seedlings (Fig. S14A) or root-only samples (Fig. 3 and S14B-E) with 0 or 4 h of 150 mM NaCl treatment. Interestingly, the induction of a set of salt stress-responsive genes in a bZIP17-dependent manner [36] was repressed in *agb1* seedlings (Fig. S14A) and root samples (Fig. 3A). These twenty-two gene expressions were compromised in *s1p-3* and *bzip17* mutant with 4-h NaCl treatment in previous studies [36, 37]. To examine whether AGB1 exercised control over these genes in roots during salt stress, their expression was assessed in *agb1* and/or *bzip17* mutants using quantitative RT-PCR (Fig. 3B-D). The seedlings exposure to 150 mM of NaCl for 4 h elevated the expression of these genes like *NAC DOMAIN CONTAINING PROTEIN 19* (*NAC019*, Fig. 3B), *MYB DOMAIN PROTEIN 75* (*MYB75*, Fig. 3C)*, RESPONSIVE TO DESICCATION 20* (*RD20*, Fig. 3D), *LIPID TRANSFER PROTEIN3* (*LTP3*, Fig. 3E)*, LATE EMBRYOGENESIS ABUNDANT PROTEIN7* (*LEA7*, Fig. 3F), *OUTER MEMBRANE TRYPTOPHAN-RICH SENSORY PROTEIN)-RELATED* (*TSPO*, Fig. 3G) in WT, but much less in *agb1* mutant. These findings fit with a model that under abiotic stresses or ABA treatment, S1P and/or S2P initiate the proteolytic activation of bZIP17, which directly upregulates genes like *NAC019* [40]*.* Next, it is worth exploring whether the downregulated gene network in *agb1* roots resulted from a group of transcription factors (TFs) regulated by AGB1. Indeed, 372 genes were down-regulated in *agb1* compared to WT under high salinity, including 18 TF genes (Fig. S14B). We then generated a Venn diagram to cover identified TFs in this study (Fig. S14C) with NaCl-induced genes in Arabidopsis roots [41] and AGB1 interactome based on the BioGrid database (https://thebiogrid.org) (Fig. S14D). Two TFs *MYB15* and *NAC069* intersected to serve as potential candidates for AGB1 to regulate expressions of salt stress-responsive gene. Indeed, both *MYB15* and *NAC069* reduced expressions in *agb1* after 4-h NaCl treatment (Fig. S14E).

### The *bZIP17* depletion genetically inhibits the *agb1* phenotype under salt stress

**Fig. 4:**
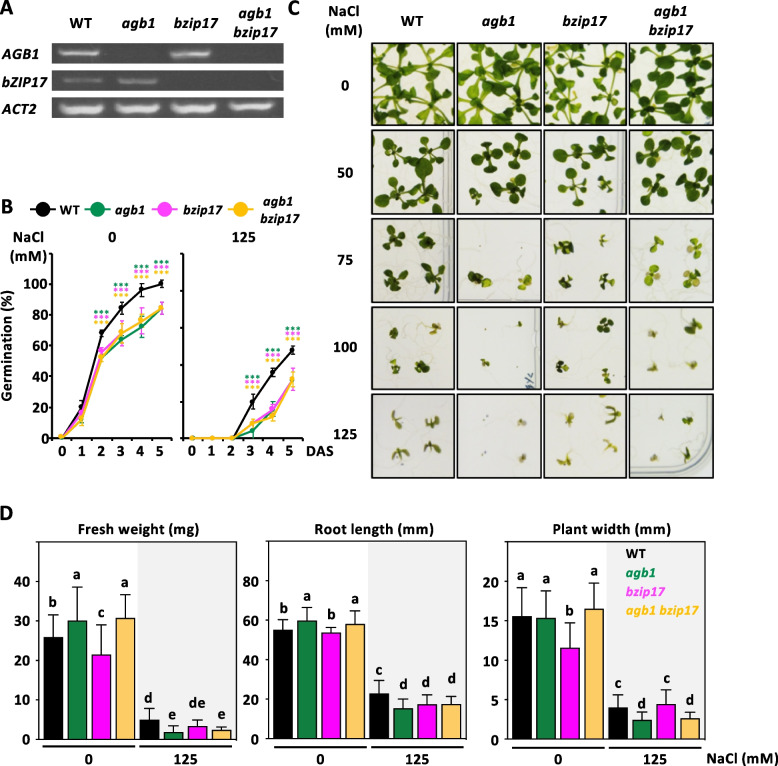
Genetic interaction of *AGB1* and *bZIP17*. **A** RT-PCR analysis for the wild type (WT), *agb1-3* (*agb1*), *bzip17-4* (*bzip17*) and *agb1 bzip17*. *ACTIN2* (*ACT2*) was used as the loading control. **B** Germination rates of *agb1* (green), *bzip17* (magenta), and *agb1 bzip17* (mustard) mutant seeds compared with the corresponding WT (black) seeds grown on MS containing 0 or 125 mM NaCl for 14 days. Each value represents the means ± SD of the germination percentage (with 25 seeds) for four independent experiments. Representative images **C** and phenotypic quantification **D** of 14-day-old WT, *agb1*, *bzip17,* and *agb1 bzip17* mutants were grown on ½ MS containing NaCl with indicated concentration to induce salt stress. **D** seedling fresh weight (left), primary root length (middle), and leave length (right) of 14-day-old seedlings grown on 0 or 125 mM NaCl shown in (D) were measured individually with three biological experiments (*n* = 25), and shown as means ± SD of 3 independent mean value. Data with different letters represent significant differences [one-way ANOVA at *P* < 0.05]

The bZIP17 and AGB1 are signaling modulators involved in various pathways, including the unfolded protein response and salt stress response. Both *agb1-2* and *bzip17* mutants exhibit hypersensitivity to salt stress [11, 36]. To investigate the genetic interactions between *AGB1* and *bZIP17*, we isolated *bzip17-4* (as *bzip17,* [42]) and generated *bzip17 pbZIP17:bZIP17* (Fig. S5, S15) and *bzip17 pbZIP17: mRFP-bZIP17* lines (Fig. S5, S16A), which displayed a complementary phenotype, rescuing the *bzip17* hypersensitive defect to salt stress (Fig. S5, S15 and S16B-E). Next, we crossed between *bzip17* and *agb1* to generate an *agb1 bzip17* double mutant, confirmed by RT-PCR analysis (Fig. 4A). Under mock conditions, *agb1 bzip17* phenocopied the *agb1* mutant, displaying lower germination rate, higher biomass, longer primary roots, and increased plant width (Fig. 4B-D). However, under high salinity, both *agb1* and *agb1 bzip17* mutants exhibited salt hypersensitivity, with smaller seedlings and shorter roots, suggesting that *agb1* is epistatic to *bzip17* (Fig. 4B-D). Furthermore, *agb1* displayed a more severe chlorotic phenotype compared to *bzip17* under the same growth conditions (Fig. 4C). Treatment of an *agb1 bzip17* double mutant with various NaCl concentrations revealed higher fresh weight compared to the *agb1* single mutant (75, 125, 150 mM NaCl, Fig. 4C, S15), indicating significant interaction effects between AGB1 and bZIP17 under salt stress. This suggests an antagonistic interaction between AGB1 and bZIP17.

### The bZIP17 and AGB1 physical interaction in vivo

**Fig. 5:**
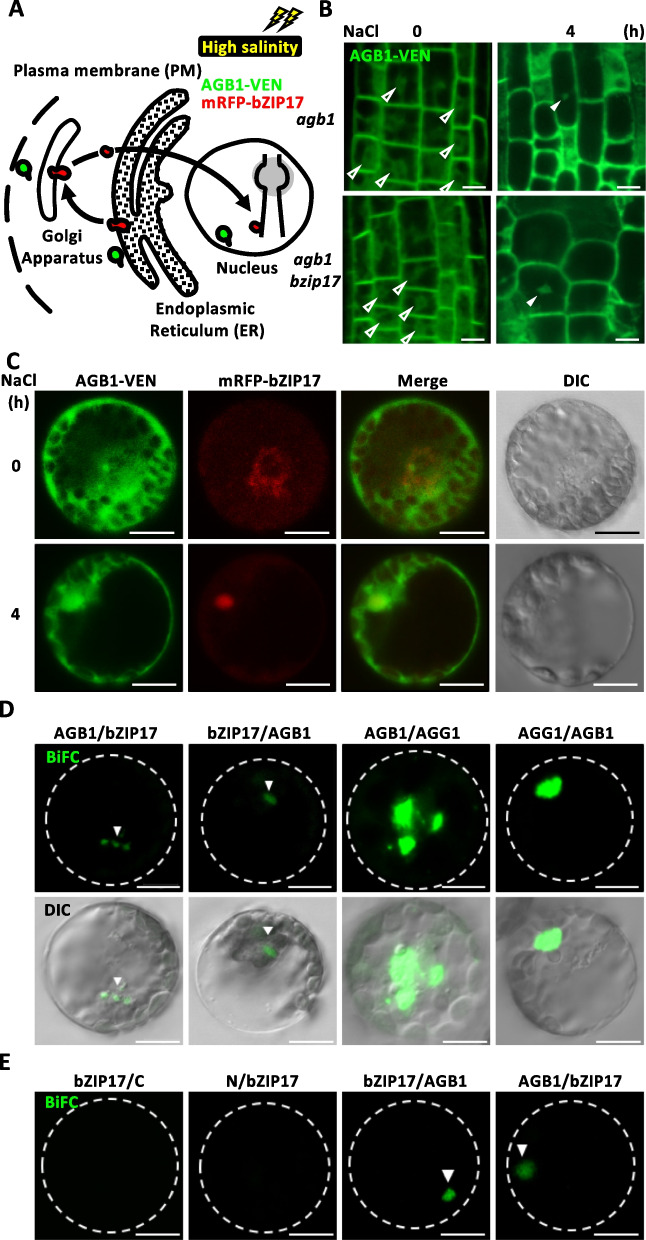
AGB1 and bZIP17 protein–protein interaction in vivo*.* **A** Schematic representation of the UPR pathway is initiated when misfolded proteins are over-accumulated in the ER lumen by activating bZIP17 under salt stress. AGB1 (*pAGB1:AGB1-VENUS*, AGB1-VEN) was marked as green pattern and bZIP17 (*pbZIP17:mRFP-bZIP17*, mRFP-bZIP17) as red pattern. **B** Subcellular localization of AGB1 in the root of 7-day-old AGB1-VEN plant in *agb1* (up) or *agb1 bzip17* (bottom) background. ER-like and nuclei localization of AGB1-VEN were marked by open or solid arrowheads, respectively. **C** AGB1-VEN and mRFP-bZIP17 were transiently expressed in WT protoplasts to observe subcellular localization after 0- or 4-h of 150 mM NaCl treatment. **D**, **E** Bimolecular Fluorescence Complementation (BiFC, green) assay for physical interaction of AGB1 and bZIP17 after 4 h of 150 mM NaCl treatment as arrowhead indicated. **D** AGG1 is a positive control for AGB1 interaction with N-YFP and C-YFP combinations. **E** A dashed circle marked protoplast boundaries. bZIP17 were paired with empty BiFC vectors (C-YFP or N-YFP) for negative control. Differential interference contrast (DIC) images to show cellular structures. Scale bars equal to 10 µm

Given the nuclear localization of AGB1 under high salinity (Fig. 2) and its involvement in initiating the bZIP17-dependent salinity stress response (Fig. 3), we next ask whether AGB1 physically interacts with bZIP17 to affect its role as a transcription factor (Fig. 5A). Under high salinity, the ER-localized bZIP17 was transferred to the Golgi apparatus, generating a spliced bZIP17 to move into the nuclei (Fig. 5C and S16F) and trigger a set of gene expressions, while the AGB1 was located in the plasma membrane, ER, and nuclei (Fig. 2, 6A). We first test whether the nuclei localization of AGB1 required bZIP17 presence in young seedlings; the AGB1-VEN showed a nuclei-like pattern under salt stress in *agb1* and *agb1 bzip17* genetic grounds, suggesting that the subcellular localization of AGB1 did not affect by the presence of bZIP17 (Fig. 5B). We next tested whether AGB1 interacts directly with bZIP17, and BiFC assays were performed in *Arabidopsis* protoplasts utilizing a pUBN/C BiFC system [43] (Fig. 5D, 5E). The coding sequences of AGB1 and AGG1 were cloned into pUBC vectors to act as positive pairs, and bZIP17 was cloned into pUBN vectors with translational fused with split YFP tag protein, respectively. No signal was observed in the negative control when the empty vector was paired with AGB1, AGG1, or bZIP17 BiFC vector. In contrast, a clear YFP signal (green) in samples expressing bZIP17 or AGG1 with AGB1 indicated interaction (Fig. 5D, 5E). These data suggests a physiological interaction between AGB1 and bZIP17 in the protoplast system.

**Fig. 6:**
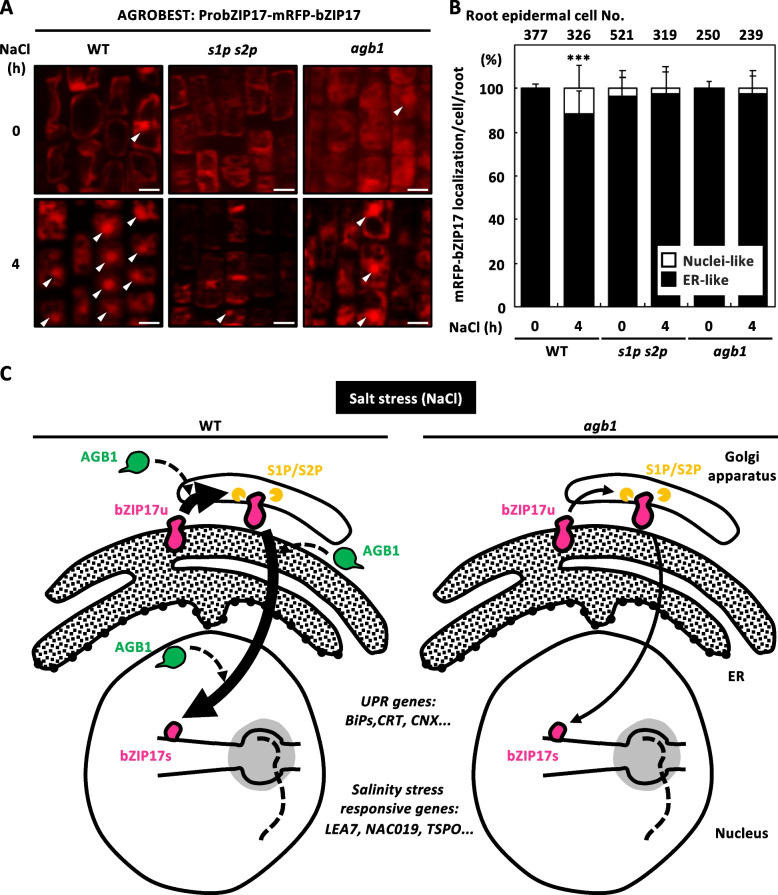
*bzip17* proteolytic processing in *agb1* mutant. **A** ARGOBEST transient expression of mRFP-bZIP17 in the 7-day-old WT, *s1p-1 s2p-1* (*s1p s2p*) and *agb1* mutant seedlings followed by 4-h of 0 or 150 mM NaCl treatment. Solid triangle marked the nuclei-like localization of mRFP-bZIP17. Scale bars equal to 10 µm. The ratio of different subcellular localization of mRFP-bZIP17 was quantified as shown in **B**. Each value represents the means ± SD of the percentage of mRFP-bZIP17 localization (ten seedlings) for three independent experiments per time point. The total numbers of examined epidermal cells for corresponding tissue were shown at the top of each bar. The epidermal cells expressed nuclei-like mRFP-bZIP17 were classified into to white group, while the ER-like mRFP-bZIP17 were classed into black group. **C** Schematic representation of AGB1-mediated salinity stress response through bZIP17 signaling. The nuclei localization of AGB1 (green) was observed after short-term salt stress treatment in the wild-type (WT) seedlings (left), and the bZIP17 (magenta)-regulated expression of salinity-responsive genes and unfolded protein responsive (UPR) genes were reduced in the absence of *AGB1*. The nuclei localization of bZIP17 through S1P/S2P-mediated proteolytic processing was also compromised in the *agb1* mutants (right)

### The repression of proteolytic processing of bZIP17 in *agb1* mutant

To test whether AGB1 functions in the S1P-S2P pathway to regulate proteolytic processing of bZIP17 for gene induction under salt stress, we first transiently expressed the biological functional mRFP-bZIP17 (Fig. 6A, 6B) in 7-day-old WT, *agb1*, or *s1p-1 s2p-1* (*s1p s2p*) seedlings by AGROBEST [44]. In the meristemic zone of the WT root epidermis, the dot-like mRFP-bZIP17 (as indicated by the white triangle) was observed in salt-treated plants but rarely seen in *s1p s2p* or *agb1* mutants (Fig. 6A). We also quantified the cells expressing nuclei-localized mRFP-bZIP17, the WT roots were significantly higher (*P* = 0.013) under salt stress (15.5 ± 7.7%) than under mock condition (1.0 ± 2.8%), whereas the nuclei-localized mRFP-bZIP17 in *agb1* or *s1p s2p* roots were not different (*agb1: P* = 0.691; *s1p s2p*: *P* = 0.486) between stressed (*agb1*: 0.0 ± 7.3%; *s1p s2p:* 3.1 ± 15.2%) and control (*agb1*: 0.0 ± 3.8%; *s1p s2p:* 4.8 ± 3.0%) conditions (Fig. 6B).

## Discussion

This study used salinity-stressed young seedlings to address a long-standing question about the functional role of AGB1 in transcriptional regulation for plant survival and fitness. It remains unknown whether the induction of a set of bZIP17-dependent salt stress-responsive genes is correlated with AGB1. We uncovered the salinity-responding nuclear localization of AGB1 among multiple tissues of young seedlings (Fig. 2). In the absence of *AGB1*, gene inductions of salt-responsive transcription factors, antioxidants, and osmoticants were compensated as *bzip17* mutant root (Fig. 3), which may contribute by the physical interaction between AGB1 and bZIP17 (Fig. 5). At the molecular level, the S1P/S2P-mediated bZIP17 proteolytic processing was abolished in *agb1* mutant to decrease the nuclear localization of bZIP17 under high salinity (Fig. 6A and 6B). At the genetic level, the *agb1 is* epistatic to *bzip17* as the *agb1 bzip17* double mutant phenocopying *agb1* but not *bzip17* (Fig. 4)*.* Therefore, our mechanistic study elucidated that AGB1 coordinated transcriptional salinity response via the regulation of bZIP17 processing and mediating the signaling pathway (Fig. 6C).

AGB1 assumes a pivotal role in responding to salt stress. The G protein interactome study identified a gene ontology term linked to the salt stress response [11]. Additionally, microarray analysis employing the *agb1* null mutant showcased increased sensitivity to salinity [19]. Phenotypic characterization of chlorotic *agb1* plants revealed reductions in chlorophyll content, fresh weight, survival rate, and stomatal aperture size [19, 21]. Furthermore, AGB1 depletion was associated with increased shoot ABA content and Na ^+^ accumulation [22, 23]. Yu and Assmann demonstrated that AGB1 physically interacts with the receptor-like kinase FERONIA (FER) and that RALF1 regulates stomatal movement through FER in a G protein-dependent manner, with AGB1, AGGs, and XLGs involved in this signaling. AGB1 and FER act additively or synergistically in salinity response, with RALF1 enhancing salt toxicity independently of AGB1, showing different genetic relationships to RALF1 in stomatal versus salinity responses [25, 45].

Under salt stress, AGB1 and bZIP17 display a functional link and epistatic interactions. Notably, they do not fully affect each other's salt-induced expression (Fig. S12) or subcellular localization (Fig. 5B and 6A). In non-stress conditions, *agb1*, *bzip17*, and *agb1 bzip17* mutants show similar germination rates (Fig. 4B). However, under stress, *bzip17* exhibits superior growth compared to *agb1* and *agb1 bzip17* in fresh weight, root length, and leaf length (Fig. 4C and 4D). For bZIP17-dependent salt-responsive gene expressions (*NAC019*, *MYB75*, *RD20*, *LTP3*, *LEA7*, and *TSPO* as shown in Fig. 3B-G), WT can induce gene expression better than *agb1*, but both *bzip17* and *agb1 bzip17* failed to do so (Fig. 3B). As Henriquez-Valencia et al. reported, during high salinity-induced ER stress, activation of the UPR pathway through transcription factor bZIP17 leads to an induction of classical chaperone marker BiP3 [46]. We measured multiple UPR expressions (*BiP3*, *BiP1/2*, *CALERTICULIN1* (*CRT1*), and *CALNEXIN* (*CNX1*)), *bzip17* mutants showed better induction than *agb1* and also *agb1 bzip17* (Fig. S13). The *agb1 bzip17* double mutant mirrors *agb1* more than *bzip17* in UPR gene expression (Fig. S12) and salt stress tolerance (Fig. 3B-G), indicating AGB1's dominant role. These results suggest that *AGB1* exerts an epistatic effect over *bZIP17*, modulating plant growth and stress responses differently and acting upstream or in conjunction with bZIP17 in these pathways.

AGB1 contributes to ER stress responses by maintaining ER homeostasis under adverse conditions such as unfolded protein accumulation [47] or environmental stressors, and interacts with ER-localized proteins involved in various cellular processes, including calcium signaling and lipid metabolism, such as RALF [25]. The bZIP17 uses posttranslational machinery to move from the ER to the Golgi apparatus, undergoing regulated intramembrane proteolysis (RIP). This process involves two Golgi-resident proteases, S1P and S2P, which cleave the transmembrane domains of bZIP17, releasing their transcription activation domains from the ER membrane [36]. The conditions of ER lumen, calcium pool, and ER membrane integrity may influence the relocation of bZIP17 from the ER to the Golgi apparatus. Additionally, AGB1 localizes to the Golgi apparatus in mammalian cells [48] and leaf epidermal cells. The transient expression of mRFP-bZIP17 driven by its promoter in *agb1* seedlings mimics the deficiency in bZIP17 relocation to the nucleus observed in *s1p s2p* mutants (Fig. 6A, 6B), suggesting that the S1P/S2P-mediated proteolytic processing may compensate in *agb1*. However, it remains unkown whether AGB1 directly affects protease activity or physically interacts with bZIP17 to isolate substrates from enzymes S1P and S2P.

Transcriptome analysis of *agb1* mutants subjected to short-term salt stress revealed increased sensitivity to elevated salinity (Fig. 3, S14), suggesting a crucial role for AGB1 in orchestrating transcriptional responses to equip seedlings with the necessary defenses for survival ([19] and the present study). Transcription factors such as bZIP17, NAC069, and MYB15 either interact with AGB1 or have their expression regulated by AGB1 (Fig. 5, S14D, S14E), making them promising candidates for further investigation into AGB1's involvement in gene expression regulation. Furthermore, during photomorphogenesis, CRY1 inhibits AGB1 to release the transcription factor HY5 (bZIP56), enabling its DNA-binding activity and promoting gene expression for hypocotyl growth [30]. AGB1 interacts with bZIP51 (VIP1) to function under drought-stress conditions [31].

### Limitations of this study

The study presents valuable insights into the role of AGB1 in salt stress responses, yet several limitations constrain it. Firstly, the lack of mechanistic understanding hinders a comprehensive elucidation of molecular pathways, such as the influence of AGB1 on S1P/S2P-mediated proteolytic cleavage of bZIP17, bZIP17 intracellular trafficking and its binding affinity to cis-elements of salt stress-responsive genes. Furthermore, focusing on a limited number of time points within a short duration may only partially capture the dynamic nature of salt stress responses over longer periods, impacting the assessment of long-term fitness. Additionally, the transcriptional analysis is confined to a subset of genes regulated by bZIP17, potentially overlooking other target genes and pathways modulated by AGB1. While BiFC assays suggest a physical interaction between AGB1 and bZIP17, further validation *in planta* is warranted. Lastly, the study's exclusive focus on *Arabidopsis thaliana* may restrict the generalizability of the findings to other crop species, underscoring the necessity for comparative studies across diverse plant species to ascertain the broader relevance of AGB1-mediated salt stress responses. Addressing these limitations through future experimental investigations will be crucial for comprehensively understanding AGB1's role in plant salt stress adaptation.

## Materials and methods

### Plant materials and growth conditions

*Arabidopsis thaliana* Columbia-0 (Col-0) ecotype was used in this study. T-DNA-tagged mutants of *agb1-3* (SALK_061896 as *agb1*), *bzip17-4* (GABI_220B01 as *bzip17* [42]), *s1p-1* (SALK_020530 as *s1p*), *s2p-1* (GABI_459C12 as *s2p*) were obtained from Arabidopsis Biological Resource Center (ABRC, http://abrc.osu.edu) and GABI-Kat (http://www.GABI-Kat.de). Seed surfaces were sterilized with 70% (v/v) ethanol and washed thrice with autoclaved ultrapure water. After one day of stratification at 4 °C, seeds were planted directly onto Petri dishes containing half-strength Murashige and Skoog (½ MS, Duchefa Biochemie, Haarlem, Nederland, M0222) medium with 0.86% (w/v) sucrose (107687; Merck, Darmstadt, Germany) and 0.6% (w/v) agar (214010; Difco, BD, MJ, USA) at pH 5.6 [49]. Plants were grown under 16 h / 8 h light–dark cycle at 22 °C with light intensity of 150 µmol m^−1^ s^−1^. Homozygous plants were isolated by PCR-based genotyping using gene-specific primers and T-DNA specific primers. The primers used were for *agb1* (KK70/KK71 and KK8/KK71), and *bzip17* (KK537/KK538 and YN1016/KK538). See supplemental Table 1 for the oligonucleotide sequences used in this study.

### Salt stress treatment, germination, and phenotypic analysis

For salt stress induction, 7-day-old seedlings were immersed in liquid MS media containing 150 mM NaCl (1.06404, Merck) for the indicated time. For long-term salt stress tolerance assay, seeds were planted on the ½ MS agar plate containing NaCl as indicated concentration for 14 days. Germination rate, seedling fresh weight (FW), primary root length, and plant width (length of two cotyledons) were measured with 11 to 25 seedlings with at least three biological replicates to quantify morphological phenotypes. The salinity tolerance analysis was performed by classifying plants according to leaf color: green, mixed (at least one white leaf), and white (all albino leaves).

### Vector construction and plant transformation

To clone the split YFP variants for bimolecular fluorescence complementation (BiFC) assay, the coding sequences of AGB1, bZIP17, and AGG1 were cloned into pENTR as pYC70, pYC75, and pYC69, respectively. For the BiFC assay, above-mentioned entry vectors were recombined into a pUBC-nYFP or pUBC-cYFP to obtain pYC73 (pUBC-AGB1-nYFP), pYC74 (pUBC-AGB1-cCFP), pYC78 (pUBN-bZIP17-nYFP), pYC79 (pUBN-bZIP17-cYFP), pYC71 (pUBC-AGG1-nYFP), pYC72 (pUBC-AGG1-cYFP).

#### *agb1 pAGB1:AGB1* (*AGB1*)

Same T3 line #5–2 was reported in [38].

#### *agb1 pAGB1:AGB1-Venus* (*AGB1-V*)

To generate an *AGB1-V* complementation line, a DNA fragment encoding triple repeats of the Venus (V) fluorescent reporter was inserted in-frame before the stop codon of the AGB1 genomic region at the *Sfo*I site into pCC87 (*pENTR-pAGB1:AGB1-Sfo*I) to express AGB1-Ven. The obtained pCC91 (*pENTR-pAGB1:AGB1-Venus*) was then recombined into a pBGW destination vector [50] using Gateway™ LR Clonase™ II (Invitrogen, Thermo Fisher Scientific, MA, USA) to obtain plasmid pCC95 (*pBGW-pAGB1:AGB1-Venus*). pCC95 was transduced into *agb1* via *Agrobacterium tumefaciens* strain GV3101 mediated transformation. Forty-eight T1 plants were selected on soil by spraying with 0.1% BASTA solution. The obtained T2 seeds were screened using BASTA and selected by genotyping. To distinguish transgenic *pAGB1:AGB1-Venus* from endogenous *AGB1*, specific primers (KK212/KK104) were designed. Line 6 was used for observation.

#### *bzip17 pbZIP17:bZIP17* (*bZIP17*)

PCR amplified the 5.8 kb of genomic sequence for *bZIP17* (*pbZIP17:bZIP17*) with primers LC125 and LC126. The fragment was cloned into the pENTR™/D_TOPO™ plasmid vector (Invitrogen) to obtain pENTR-ProbZIP17:bZIP17, which was then recombined into the pBGW destination vector [50] with the use of Gateway™ LR Clonase™ II (Invitrogen) to obtain pLC25 (pBGW-ProbZIP17:bZIP17). pLC25 was transduced into *bzip17* via *A. tumefaciens* strain GV3101 mediated transformation. In total, 24 T1 plants were selected on soil by spraying with 0.1% BASTA solution. The obtained T2 seeds were screened using BASTA and selected by genotyping. Lines 3, 5, 7 and 18 were used for observation, and Line 18 was selected as a representative line.

#### *bzip17 pbZIP17: mRFP-bZIP17* (*m-bZIP17*)

To generate an *m-bZIP17* complementation line, a DNA fragment encoding mRFP fluorescent reporter was inserted in-frame after the start codon of the bZIP17 coding sequences at the *Sfo*I site into *pENTR-pbZIP17: Sfo*I*-bZIP17* to express mRFP-bZIP17. The obtained pYC87 (*pENTR-pbZIP17: mRFP-bZIP17*) was then recombined into a pKGW destination vector [50] using Gateway™ LR Clonase™ II (Invitrogen, Thermo Fisher Scientific, MA, USA) to obtain plasmid pYC89 (*pKGW-pbZIP17: mRFP-bZIP17*). In total, 24 T1 plants were selected on ½ MS agar plate containing the antibiotic Kanamycin. The obtained T2 seeds were screened using Kanamycin and selected by PCR-based genotyping. To distinguish transgenic *pbZIP17: mRFP-bZIP17* from endogenous *bZIP17*, specific primers (LC12/KK418) were designed. Lines 16, 17, and 18 were used for observation, and Line 16 was selected as a representative line.

### Confocal laser-microscopy observation

For the transient expression assay, a drop of transformed protoplasts was applied onto a glass slide with a ring sticker. The fluorescent signals were observed under confocal laser-scanning microscopy (LSM 510 Meta; Carl Zeiss, Jena, Germany) equipped with a C-Apochromat × 63 objective with a 1.2 numerical aperture. For live imaging in primary root, hypocotyl, and cotyledon, Venus or mRFP fluorescences in 7-day-old *AGB1-V* or *m-bZIP17* seedlings, respectively, were observed under a microscope equipped with a C-Apochromat × 40 objective with 1.2 numerical aperture. For plasma membrane staining, seedlings were immersed in 10 μg/ml of FM4-64 (Invitrogen™ 13320) for 3 min. For ER staining, samples were immersed in 1 μM of ER-tracker™ Blue-White DPX (Invitrogen™ E12353) for 5 min. For nuclei staining, root samples were immersed in 10 μg/ml of DAPI (Thermo Fisher Scientific 62247). After staining the samples were observed under confocal microscopy, and images were captured using an LSM 510 v3.2 (Carl Zeiss) with filters for DAPI or ER-tracker Blue-White DPX (Diode 405 nm laser, band-pass 420–480 nm); Venus or YFP (Argon 514 nm laser, band-pass 520–555 nm); FM 4–64 or mRFP (HeNe 543 nm laser, band-pass 560–615 nm).

### Immunoblotting and nuclei isolation

Ten of 7-day-old seedlings were homogenized in 100 µl lysis buffer [50 mM Tris HCl (Merck 648,317), pH 6.8, 2% (w/v) SDS (Merck 822050), 10 mM 2-mercaptoethanol (2-ME, Merck 805740), 1% (v/v) protease inhibitor cocktail (Sigma-Aldrich P9599). The homogenate was stood on ice for 20 min and centrifuged at 16,000 g for 10 min at 4 °C. The supernatant (100 µl) was added to 2 × sample buffer (50 mM Tris-HCl, pH 6.8, 10% (w/v) SDS, 10%(v/v) 2-ME, 526 mM sucrose, 0.1% (w/v) bromophenol blue (Merck 108122)). Samples were boiled for 3 min at 95 °C and loaded and separated by 10% SDS-PAGE, blotted onto 0.45 µm PVDF blotting membrane (10600023; GE Healthcare, PA, USA) and probed with primary and secondary antibodies, as follows: rabbit polyclonal anti-GFP (for AGB1-VEN, 1:3,000, Invitrogen™ A-11122), mouse monoclonal anti-Actin (1:5,000, Agrisera AS10-702), rabbit polyclonal anti-Histone H3 (1:3,000, Agrisera AS10-710), goat anti-rabbit IgG-HRP (1:10,000, Abcam ab6721) and goat anti-mouse IgG-HRP (1:10,000, Abcam). The target proteins were visualized by use of Image Quant LAS4000 (GE Health). For nuclei isolation, CelLytic™ PN Isolation/Extraction Kit (Sigma CELLYTPN1) was used according to the manufacturer’s instructions with minor modifications. In brief, 1 g of fresh weight (~ 700 of 7-day-old seedlings) were homogenized in 3 ml of NIBA buffer [25% (v/v) 4 × Nuclei Isolation Buffer (Sigma-Aldrich N8304), 10 mM Dithiothreitol Merck #805740), 1% (v/v) protease inhibitor cocktail (Sigma-Aldrich P9599)]. The homogenate was filtrated through the Miracloth (Merck) as a total fraction and then centrifuged at 1,300 g for 10 min at 4 °C. The resulting supernatant was kept as a cytosolic fraction, and the pellet was resuspended by NIBA buffer containing 0.3% Triton X-100. After 30 min incubation at 4 °C, the resuspension was centrifuged at 12,000 g for 5 min at 4 °C with three repeats. The resulting pellet was resuspended by 1 × SDS sample buffer and incubated at 95 °C for 3 min. After being centrifuged at 12,000 g for 5 min at 4 °C, the supernatant was kept as a nucleus fraction.

### Microarray analysis

For salt stress treatment, 7-day-old Arabidopsis seedlings were subjected to ½ MS liquid medium containing 0 (control) or 150 mM NaCl (stress) for 4 h, for root samples were dissected right after salt stress treatment and harvested in liquid nitrogen. Total RNA was extracted using RNeasy Plant mini kit according to the manufacturer’s instructions (Qiagen) with in-membrane digestion of DNase (Qiagen) to remove genomic DNA contamination and quantified by 260/280 nm UV light absorption. For quality control, the integrity of RNA was determined by Agilent Bioanalyzer 2100 (Agilent Technologies, Palo Alto, CA, USA). Total RNA was amplified by a Low Input Quick-Amp Labeling kit (Agilent Technologies). Preparation of fluorescence-labelled cDNA and microarray experiments were performed at the DNA Microarray Core Facility, Institute of Plant and Microbial Biology, Academia Sinica, Taiwan. Agilent Arabidopsis (V4) Gene Expression Microarray 4 × 44k chips were used in this study. Labelling of cDNA probes and hybridization experiments were performed according to the single-colour microarray protocols provided by the manufacturer. The Agilent DNA Microarray Scanner G2565CA and Agilent Feature Extraction 10.7.1.1 software detected the fluorescence signals. Three independent biological replicates were conducted using cDNA from control and stress samples.

### RNA preparation, cDNA synthesis and reverse-transcription PCR (RT-PCR), quantitative RT-PCR

Seedlings were frozen by immersion in liquid nitrogen and stored at -80 °C until use. Total RNA was extracted using a standard TRI reagent solution (Invitrogen AM9738). In brief, ten of 7-day-old seedlings were homogenized in 600 µl of TRI reagent, followed by a phase separation step with 120 µl chloroform (Merck 107024). RNA was precipitated with 300 µl isopropanol (Merck 107022) and then 0.3 M sodium acetate (Merck 106268), washed with ethanol and resuspended in 30 µl of diethylpyrocarbonate (DEPC, Merck 298711) -treated water. Genomic contamination was removed using RNase-free DNase set (Qiagen, Hilden, Germany) according to the manufacturer’s instructions. Five hundred ng RNA was used for complementary DNA (cDNA) synthesis by the SuperScript III First-Strand Synthesis SuperMix (Invitrogen 1172050). Fifty ng cDNA was used as template for quantitative RT-PCR with SYBR^TM^ Green PCR Master Mix (Applied Biosystems™ Thermo Fisher Scientific) detection and performed in triplicate using the Applied Biosystems 7500 fast real-time PCR system. Data were analyzed by the comparative threshold cycle method (ΔΔCT methods). The transcript level was normalized to the *ACTIN2* gene (*ACT2*, KK129/KK130) for each sample. For *LIPID TRANSFER PROTEIN 3 (LTP3, YC114/YC115)*, *RESPONSIVE TO DESICCATION 20* (*RD20*, YC116/YC117), *NAC DOMAIN CONTAINING PROTEIN 19* (*NAC019*, YC32/YC33), *MYB DOMAIN 75 (MYB75, YC64/YC65), LATE EMBRYOGENESIS ABUNDANT 7 (LEA7, YC120/YC121)* and *TSPO* (YC118/YC119), the relative transcript level is expressed as the fold change (mean ± SD) in each genotype under mock (0 mM NaCl) or salt (150 mM NaCl) treatment relative to the mock control in the wild type (set to value as 1) from three biological replicates with three technical replicates. The primer sets for quantitative RT-PCR are listed in supplemental Table 1.

### Protoplast isolation and BiFC assay

Protoplasts were isolated from 20- to 22-day-old Arabidopsis WT leaves using fungal cellulase (1% (v/v) ‘Onozuka’ R10, Yakult, Tokyo, Japan) and macerozyme (‘Onozuka’ R10, Yakult) to remove cell walls accordingly with minor modification [51]. DNA transfection was performed using the PEG-calcium solution, followed by 16-h incubation at 24°C. Transformed protoplasts were observed under a laser-scanning confocal microscope.

### AGROBEST transient expression

For a transient expression to observe the bZIP17 processing in seedlings, the AGROBEST method was used with minor modifications [44]. In Brief, seeds were germinated in the MS liquid media. Three-day-old WT, *agb1* and *s1p s2p* mutant seedlings were infected with *Agrobacteria tumefaciens* strain C58C1 (pTiB6S3ΔT)^H^ carrying the pYC89 (pKGW-ProbZIP17: mRFP-bZIP17) in ABM-MS [½ AB-MES, ¼ MS, 0.25% (w/v) sucrose, pH5.5] liquid medium for 2 days. The co-cultivation medium was then replaced with 1 ml fresh ½ MS medium and then incubated for 2 days. For salt stress assay, seedlings were incubated in ½ MS medium containing 0 or 150 mM NaCl for 4 h and then observed the mRFP-bZIP17 signals under confocal microscopy.

## Accession Numbers

*AGB1* (*At4g34460*), *bZIP17* (*At2g40950*), *AGG1* (*AT3G63420*).

## Conclusions

In conclusion, this study elucidates the vital role of AGB1 in plant salt stress responses through its interaction with the transcription factor bZIP17 and its regulation of crucial stress-responsive pathways. The findings demonstrate that AGB1's subcellular localization changes under salt stress are essential for initiating specific gene expressions necessary for plant survival. Genetic analyses further highlight the sensitivity of *AGB1* mutants to salt stress, emphasizing AGB1's importance in cellular homeostasis and overall plant fitness. This research contributes to our understanding of plant stress mechanisms and opens possibilities for developing crops with improved tolerance to salinity, addressing agricultural challenges posed by environmental stresses. However, more detailed studies are required to fully explore the implications of these mechanisms across various plant species and conditions.

### Supplementary Information

Supplementary Material 1.

Supplementary Material 2.

## Data Availability

The datasets generated and analyzed in this study are available from the Gene Expression Omnibus (GEO) under accession number GSE264404.

## Abbreviations

ABA: Abscisic acid
ABRE: Abscisic acid-responsive element binding protein
AGB1: GTP-BINDING PROTEIN BETA 1
AGG: G-PROTEIN GAMMA-SUBUNIT
BBX21: B-BOX DOMAIN PROTEIN 21
BES1: BRI1-EMS-SUPPRESSOR 1
BiFC: Bimolecular fluorescence complementation
bZIP: Basic leucine zipper
CRY1: CRYPTOCHROME1
FER: FERONIA
G protein: Heterotrimeric guanine nucleotide-binding protein
GPCR: G-protein-coupled receptor
GPA1: G PROTEIN ALPHA SUBUNIT 1
HY5: ELONGATED HYPOCOTYL 5
LEA: LATE EMBRYOGENESIS ABUNDANT
MYB: Myeloblastosis
MPK6: MAP KINASE 6
mRFP: Monomeric Red Fluorescent Protein
NAC: NAM, ATAF1/2, and CUC2
PHYB: PHYTOCHROME B
PIF3: PHYTOCHROME INTERACTING FACTOR 3
RALF1: RAPID ALKALINIZATION FACTOR 1
RD20: RESPONSIVE TO DESICCATION 20
ROS: Reactive oxygen species
S1P: SITE-1 PROTEASE
S2P: SITE-2 PROTEASE
SOS: SALT OVERLY SENSITIVE
TSPO: OUTER MEMBRANE TRYPTOPHAN-RICH SENSORY PROTEIN-RELATED
UB: Ubiquitin
VIP1: VIRE2-INTERACTING PROTEIN 1
XLG: EXTRA LARGE G-PROTEIN
YFP: Yellow fluorescent protein
