# Supplementary material for: Arabidopsis AGB1 participates in salinity response through bZIP17-mediated unfolded protein response
Source: BMC Plant Biol. 2024 Jun 21;24:586. doi: 10.1186/s12870-024-05296-x (PMC11191249; doi:10.1186/s12870-024-05296-x)
Supplement: Supplementary file 1 — Supplementary Material 1. [file 12870_2024_5296_MOESM1_ESM.pdf]

Figure S1

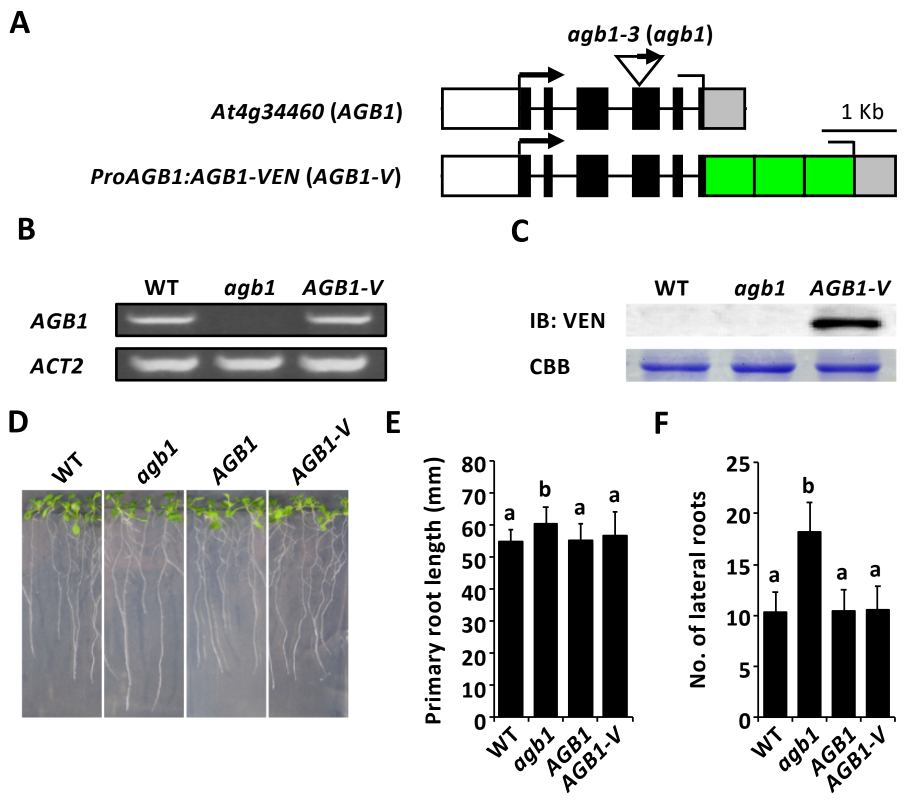

Supplemental Figure 1. *AGB1* genomic DNA fragment with Venus tag complements *agb1* defects.
(**A**) The gene structure of the *AGB1* gene (*At4g34460.1*) and its mutant *(agb1-3, agb1)* generated by T-DNA insertion. White box as the promoter, black boxes as six exons, black line as five introns, gray box as 3'UTR and green boxes as triplicate Venus. (**B**) RT-PCR analysis for the wild type (WT), *agb1-3* (*agb1*), *agb1 pAGB1:AGB1-Venus* (*AGB1-V*). *ACTIN2* (*ACT2*) was used as positive control. (**C**) Immunoblot analysis of AGB1-VEN proteins. CBB staining as the loading control. (**D**) Representative images of vertical growth of 14-day WT, *agb1*, *agb1 pAGB1:AGB1* (*AGB1*) and *AGB1-V* seedlings on ½ MS agar plates. The root phenotype including primary root length (**E**) and lateral root number (**F**) were shown as bar chart.

Figure S2

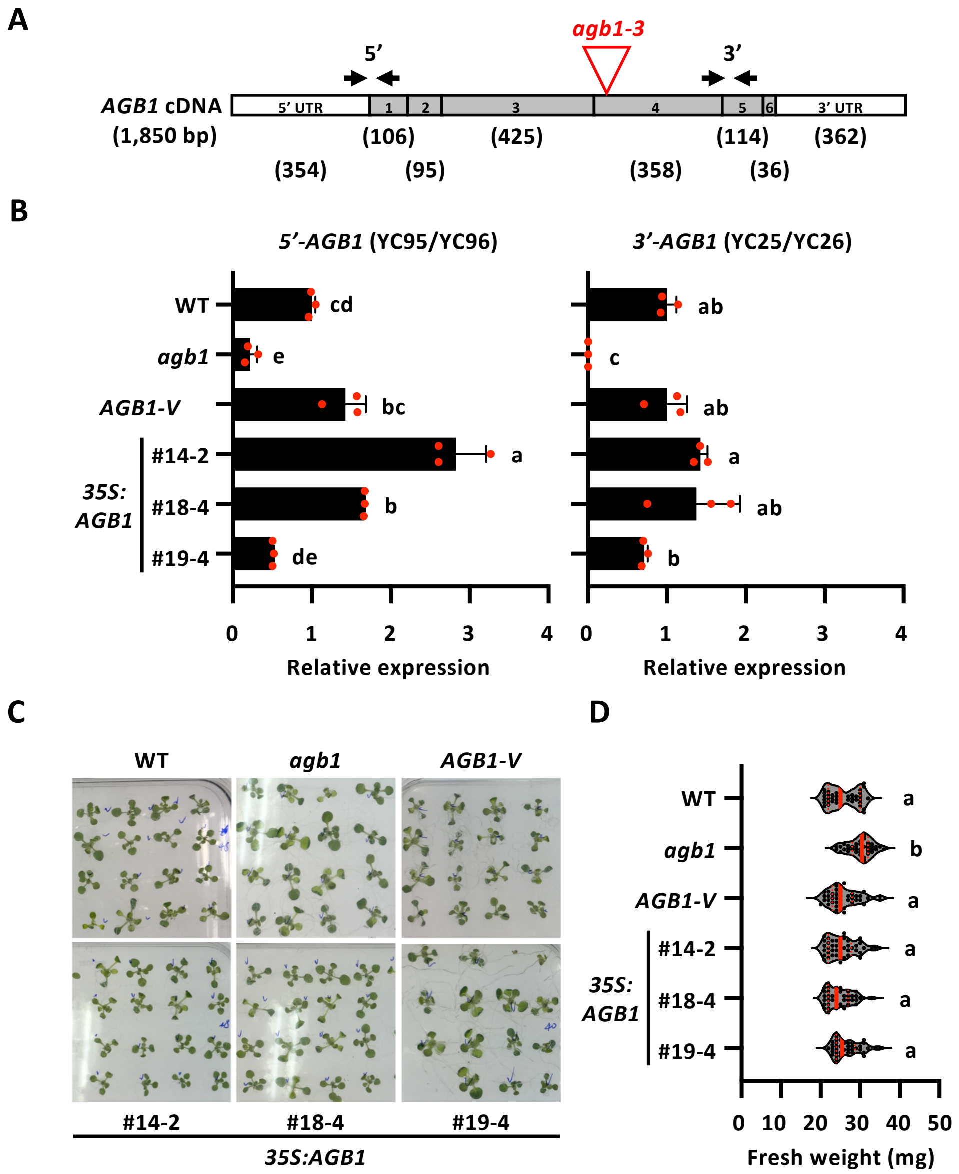

Supplemental Figure 2. The transgenic *AGB1* overexpression lines rescue growth phenotype in *agb1.* (**A**) Schematic representation of *AGB1* (At4g34460). White boxes represent untranslated regions (UTR). Gray boxes represent exons, and the positions of the T-DNA insertions in *agb1-3* are shown. Arrows indicate the positions of oligonucleotide primers used for the quantitative RT-PCR analysis in (**B**). The length of each fragment was noted inside of the parentheses below. (**B**) Relative expression level of *AGB1* in the 7-day-old wild type (WT), *agb1* mutant, complementation line *AGB1-V,* and three independent transgenic seedlings harboring *p35S:AGB1* (lines #14-2, #18-4, and #19-4) in Col-0 genetic background by quantitative RT-PCR analysis. Data are shown from three biologically independent experiments with three technical replicates. Representative images (**C**) and fresh weight measurement (**D**) of 14-d-old WT, *agb1* mutant, transgenic *AGB1-V* and *p35S:AGB1* plants were grown on 1/2 MS. (**D**) The fresh weight was measured individually with three biological experiments (n=40), and shown as violin plots with all points. Data with different letters represent significant differences [one-way ANOVA at $P < 0.05$]

Figure S3

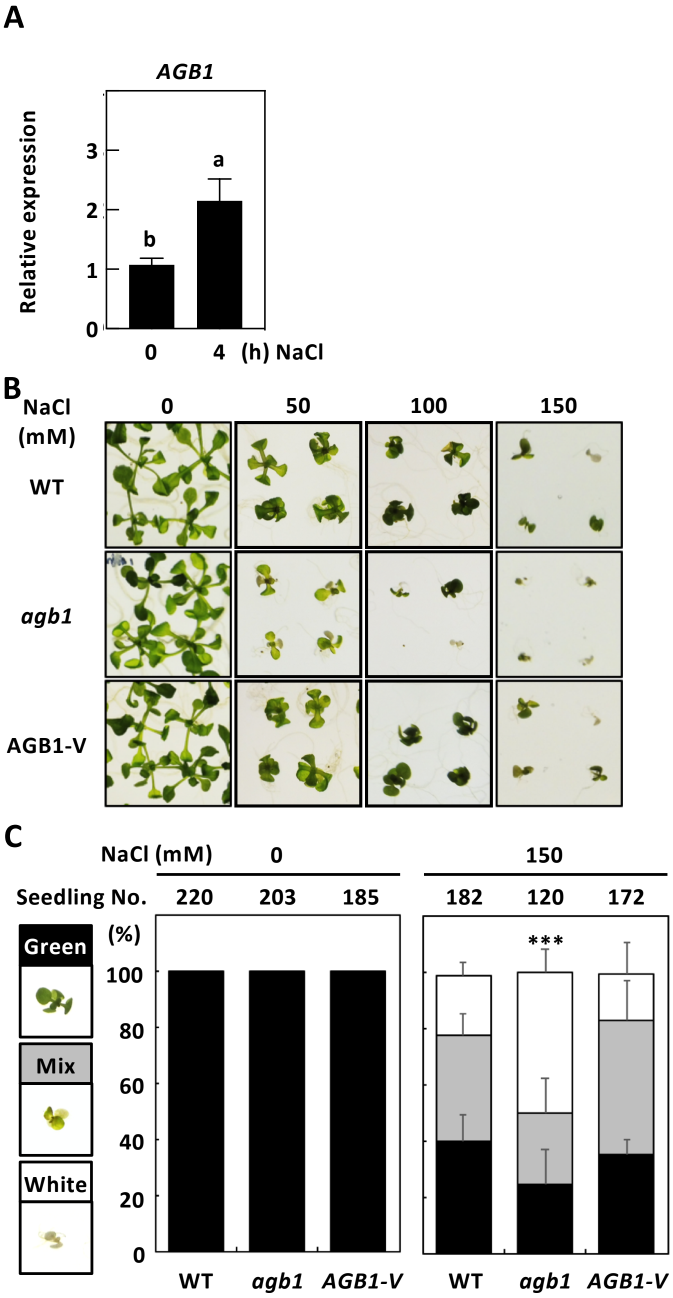

Supplemental Figure 3. *AGB1-V* is biological functioned for seedling fitness under high salinity.
(**A**) Quantitative RT-PCR analysis of 7-day-old wild-type (WT) seedlings with 0 or 4 h of 150 mM NaCl treatment. The *AGB1* expressions were normalized to *ACTIN2* and average *AGB1* expression level under normal condition was set as 1. Data were mean ± SD of three biological replicates. Data with different letters represent significant differences [one-way ANOVA at P < 0.05]. (**B**) Representative images of 14-day-old WT, *agb1* mutant, and *agb1 pAGB1:AGB1-Venus* (*AGB1-V*) transgenic plants were grown on 1/2 MS containing NaCl with indicated concentration to induce salt stress. (**C**) The morphological phenotypes were classified into healthy (Green, black box), at least one *albino* leaf (Mix, gray box) and all *albino* leaves (White , white box) groups and analyzed for their salt tolerance. Number of seedlings belong different groups were shown above each bar. Error bar, Mean + SD. Asterisk indicated the statistically differences comparing to WT. ***, *P*<0.001.

Figure S4

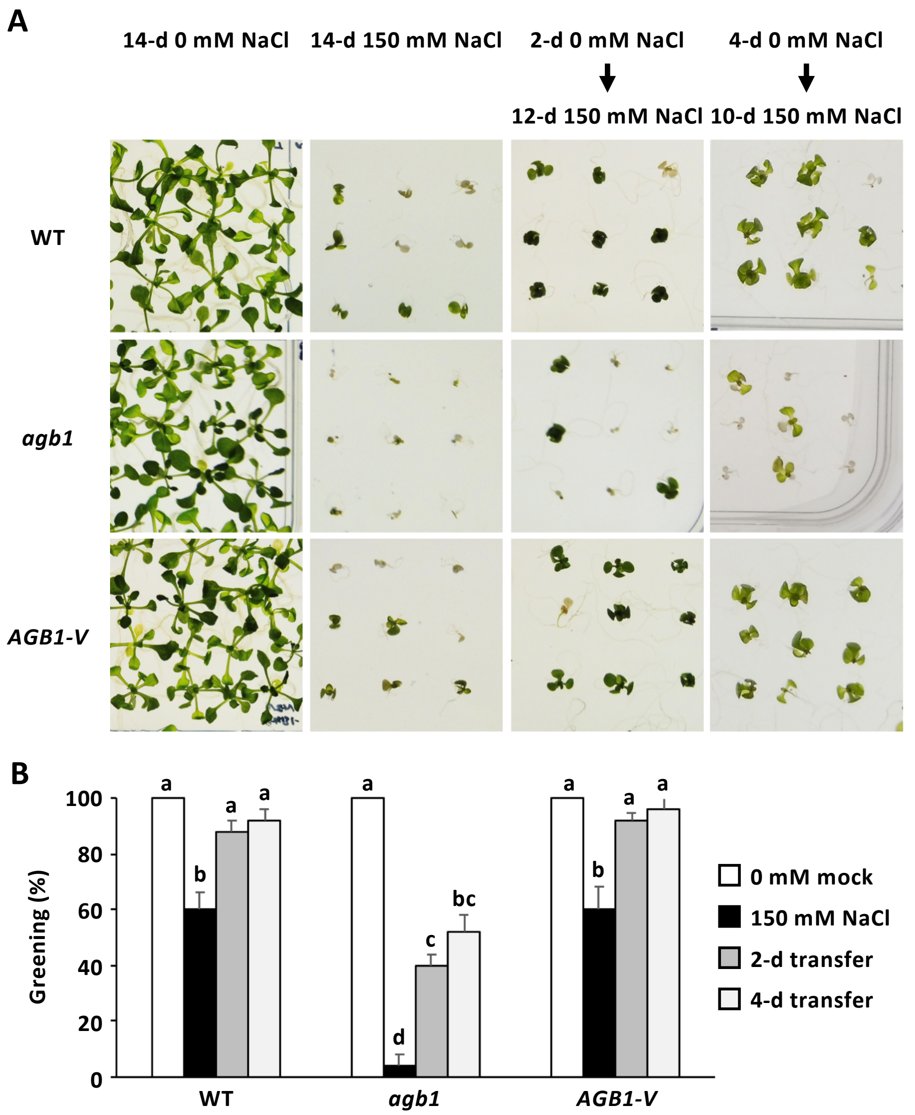

Supplemental Figure 4. The seed germination under high salinity and recovery after transferring to normal conditions.

(**A**) Representative images of 14-day-old wild type (WT), *agb1* mutant, and *agb1 pAGB1:AGB1-Venus* (AGB1-V) transgenic plants were grown on 1/2 MS containing NaCl with indicated concentration to induce salt stress, or transferred from ½ MS agar plate to 150 mM NaCl containing plate after 2-d or 4-d. (**B**) The greening phenotype was counted for the green seedlings versus total number per condition and showed as percentages. Continuous grown on 0 mM NaCl (white), or 150 mM NaCl (black), and 2-day (dark grey) or 4-day (light grey) grown on 0 mM NaCl and then transfer to 150 mM NaCl until 14 days (n=25). Error bar, Mean + SD. Data with different letters represent significant b [one-way ANOVA at $P < 0.05$].

Figure S5

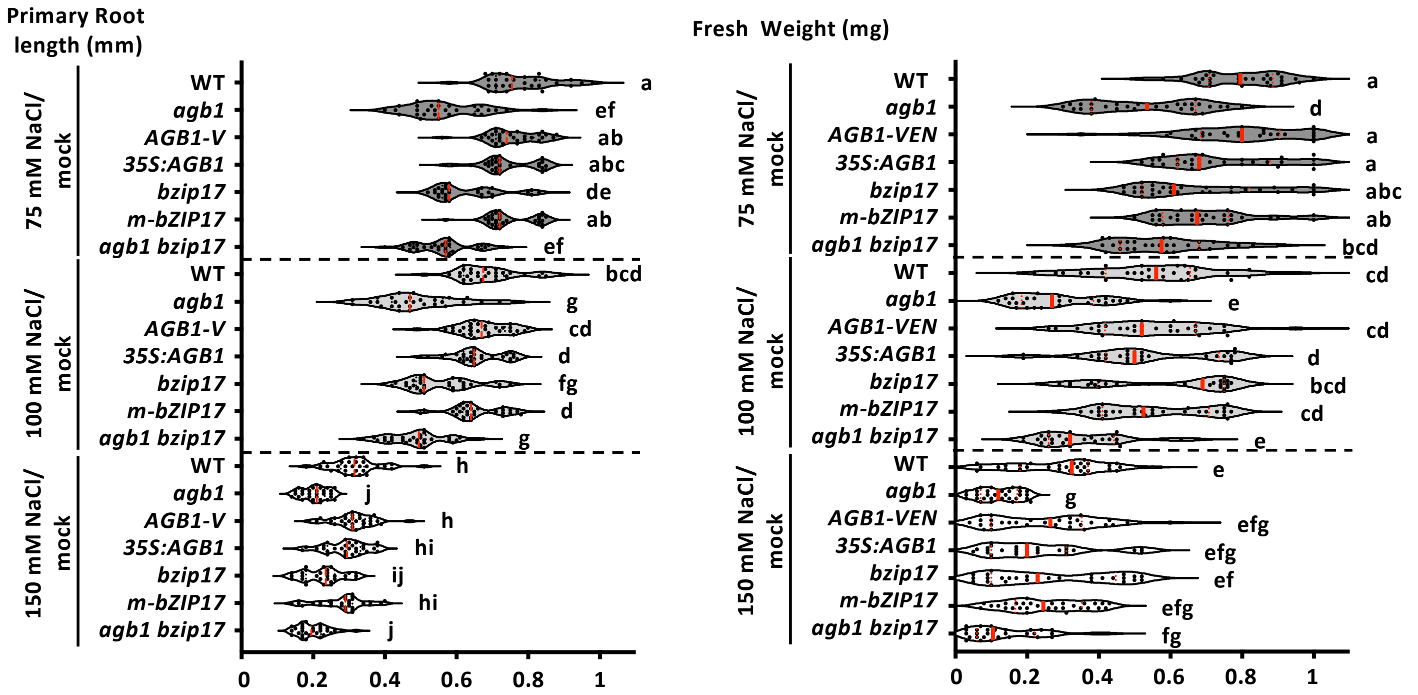

Supplemental Figure 5. Salt stress tolerance index (STI) of 14-day-old seedlings under various salinity conditions.

The primary root length (**A**) or fresh weight (**B**) of 14-day-old wild-type (WT), *agb1-3 (agb1), agb1 pAGB1:AGB1-Venus* (*AGB1-V*), *Col-0 p35S:AGB1 (35S:AGB1)*, *bzip17-4* (*bzip17*), *bzip17 pbZIP17:mRFP-bZIP17* (*mRFP-bZIP17*), and *agb1 bzip17* seedlings were quantified to calculate the ratio of the plant subjected to 75 (dark grey), 100 (light grey), or 150 mM (white) NaCl treatment relative to the plant of mock (0 mM NaCl). The STI was shown as violin plots with all points (n=30). Data with different letters represent significant [one-way ANOVA at P < 0.05].

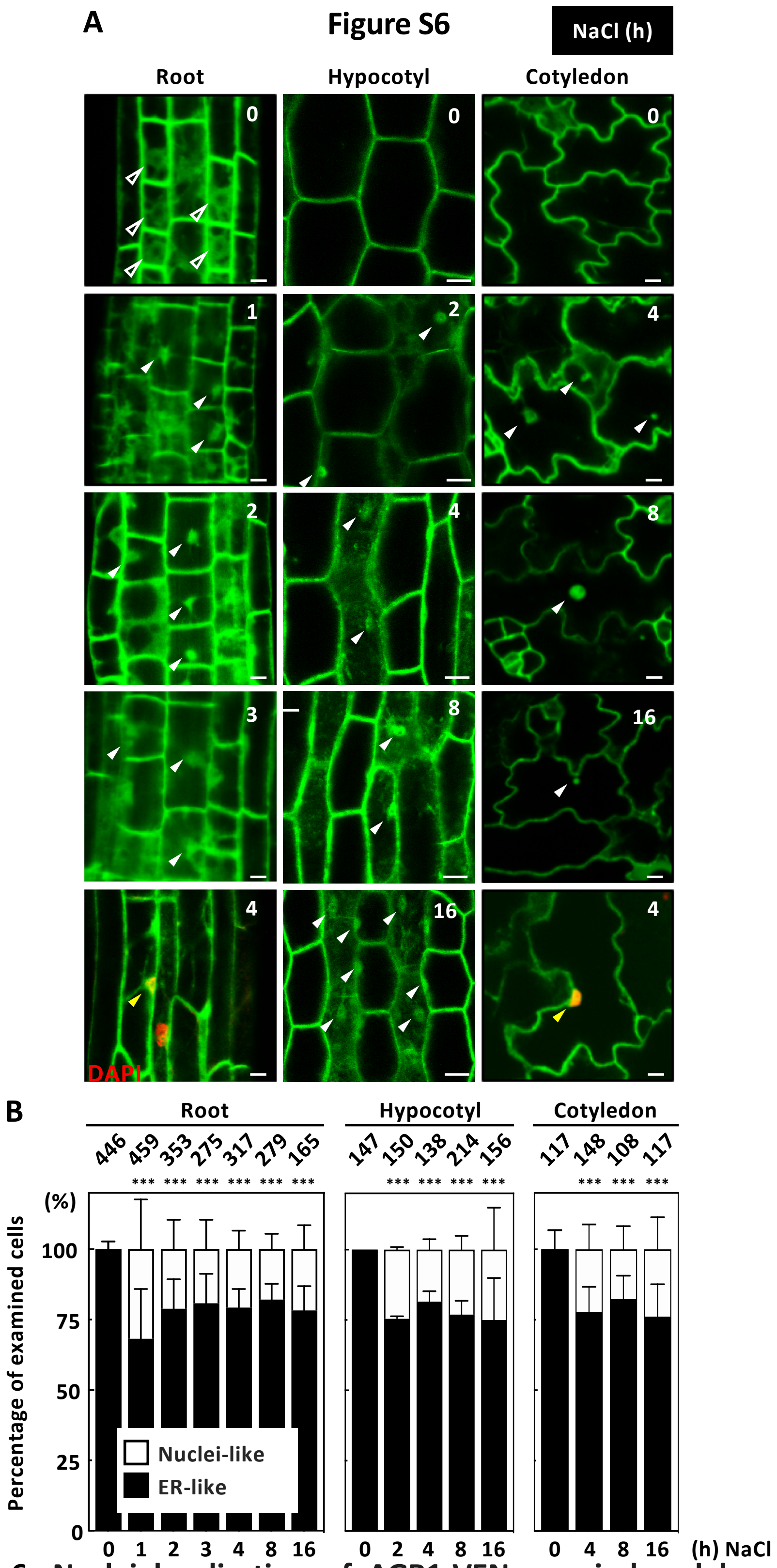

Supplemental Figure 6. Nuclei localization of AGB1-VEN was induced by salt stress in whole seedling.

(**A**) Representative images of root, stem and leaf epidermis of pAGB1:AGB1-VENUS (AGB1-VEN) in a stable transgenic complementation plant in the absence or presence of 150 mM NaCl containing ½ MS liquid medium as indicated time for salt stress induction. Solid arrow heads indicated the ER-like AGB1 localization while the empty white arrow heads indicated the nuclei localization of AGB1. The nulcei localization of AGB1 was confirmed by DAPI staining (red). The ratio of different subcellular localization of AGB1-VEN were quantified as shown in (**B**). Scale bars equal to 10 μm. (**B**) Quantification analysis of subcellular localization of AGB1 in root, hypocotyl and cotyledon of 7-day-old *AGB1-V* plant. Each value represents the means ± SD of the percentage of AGB1-VEN localization (ten seedlings) for at three independent experiments per time point. The total numbers of examined epidermal cells for corresponding tissue were shown at the top of each bar. The epidermal cells expressed nuclei-like AGB1-VEN were classified into to white group, while the ER-like AGB1-VEN were classed into black group. Data with different letters represent significant [one-way ANOVA at $P < 0.05$].

Figure S7

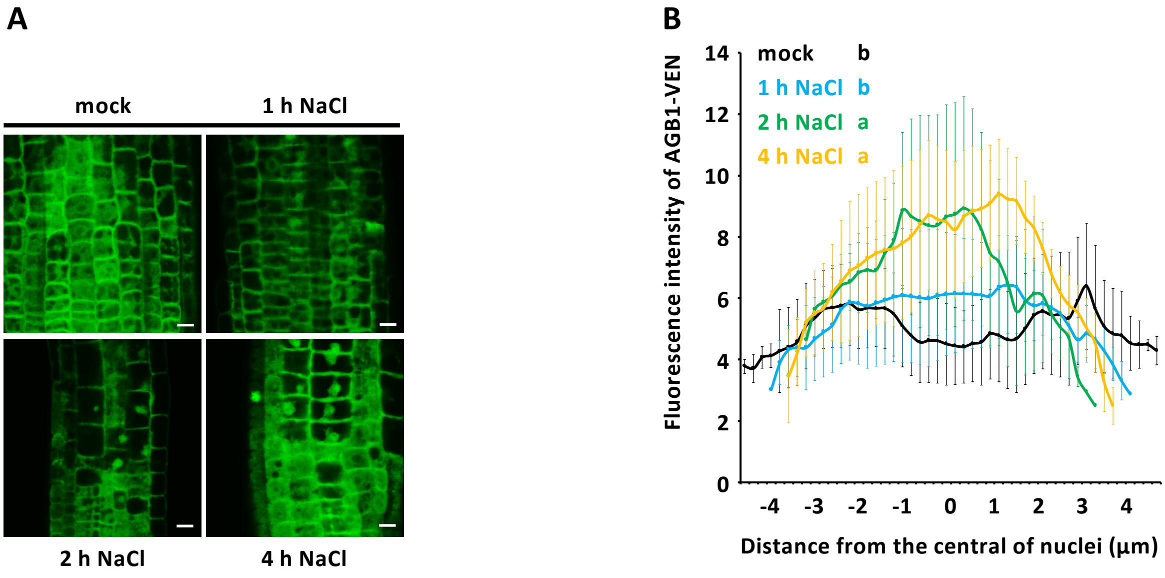

Supplemental Figure 7. The distribution of fluorescent signals of AGB1-VEN in the root epidermal cell of meristemic zone.

(**A**) Representative images of root epidermis of AGB1-VEN in a stable transgenic complementation plant *(AGB1-V)* in the absence (1/2 MS medium, 0 h) or presence of 150 mM NaCl as indicated time for salt stress induction . Scale bars equal to 10 μm. (**B**) Quantification analysis of fluorescence intensity of AGB1-Venus in the root epidermis of 7-day-old *AGB1-V* plant under various conditions including mock (black), 1-h (blue), 2-h (green), 4-h (yellow) of 150 mM NaCl treatment. The line charts were generated using the median AGB1-VEN intensity across the diameter of epidermal cells (n=30) on the y-axis, with the distance from the center of the nuclei plotted on the x-axis. Each value represents the mean ± SD (n=30). Data with different letters represent significant [one-way ANOVA at $P < 0.05$].

Figure S8

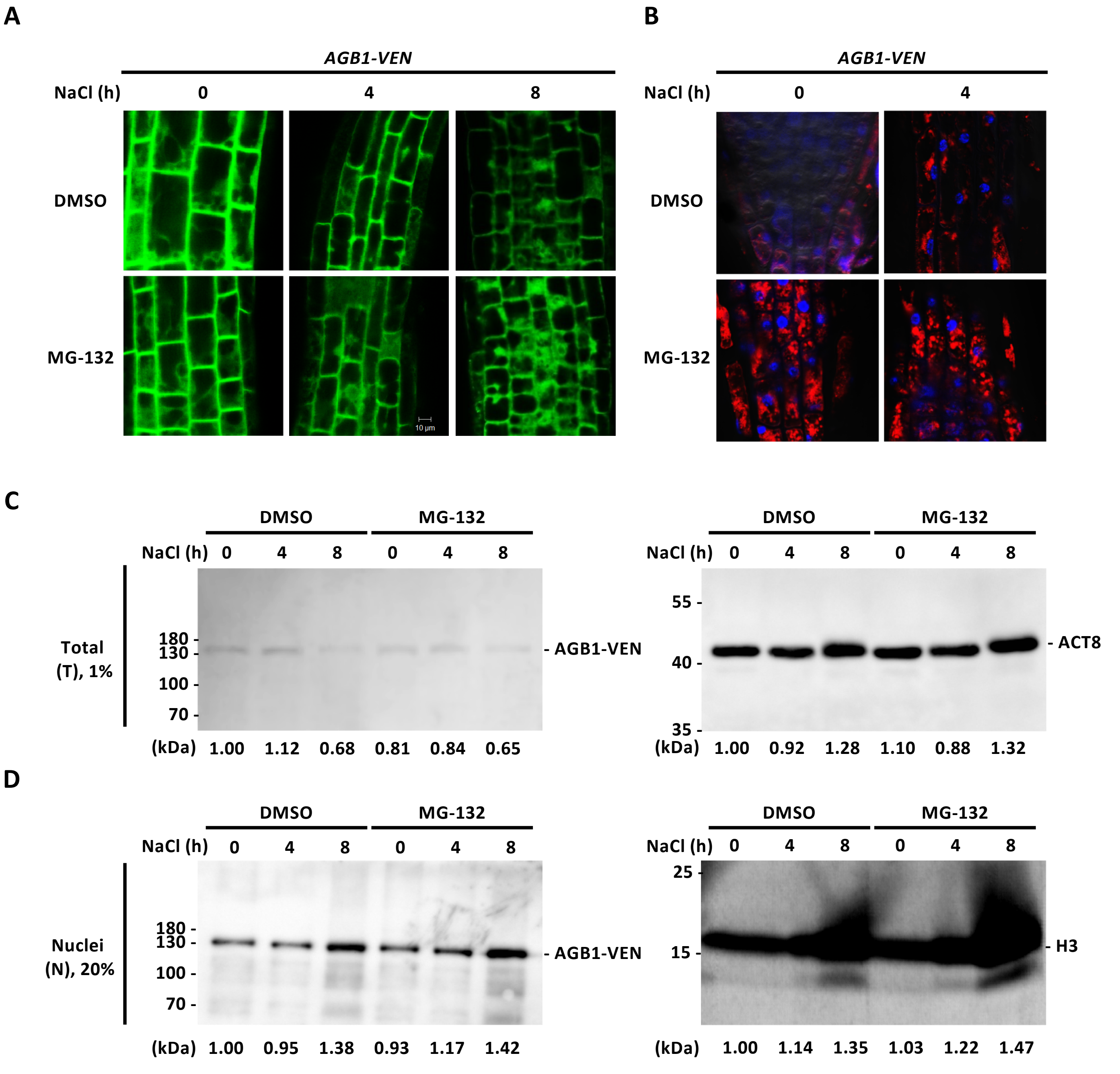

Supplemental Figure 8. The protein turnover of nuclei-localized AGB1-VEN under salt stress.
The 7-d-old of *agb1 AGB1-V* seedlings treated with 0 or 150 mM NaCl containing 1/2 MS liquid media with addition of DMSO or 10 μM MG-132 for 2, 4 or 8 h according to indicated time. (**A**) Representative images of the subcellular localization of AGB1-VEN in the root epidermis. (**B**) The misfolded proteins were detected by Aggresome dye ProteoStat (Red), the nuclei localizations were confirmed by DAPI staining (Blue). The total (**C**) and nuclei fractions (**D**) were subjected to immunoblots against GFP (left), ACTIN8 for cytosolic fractions (right panel in **C**), and Histone H3 for nuclear fractions (right panel in **D**).

Figure S9

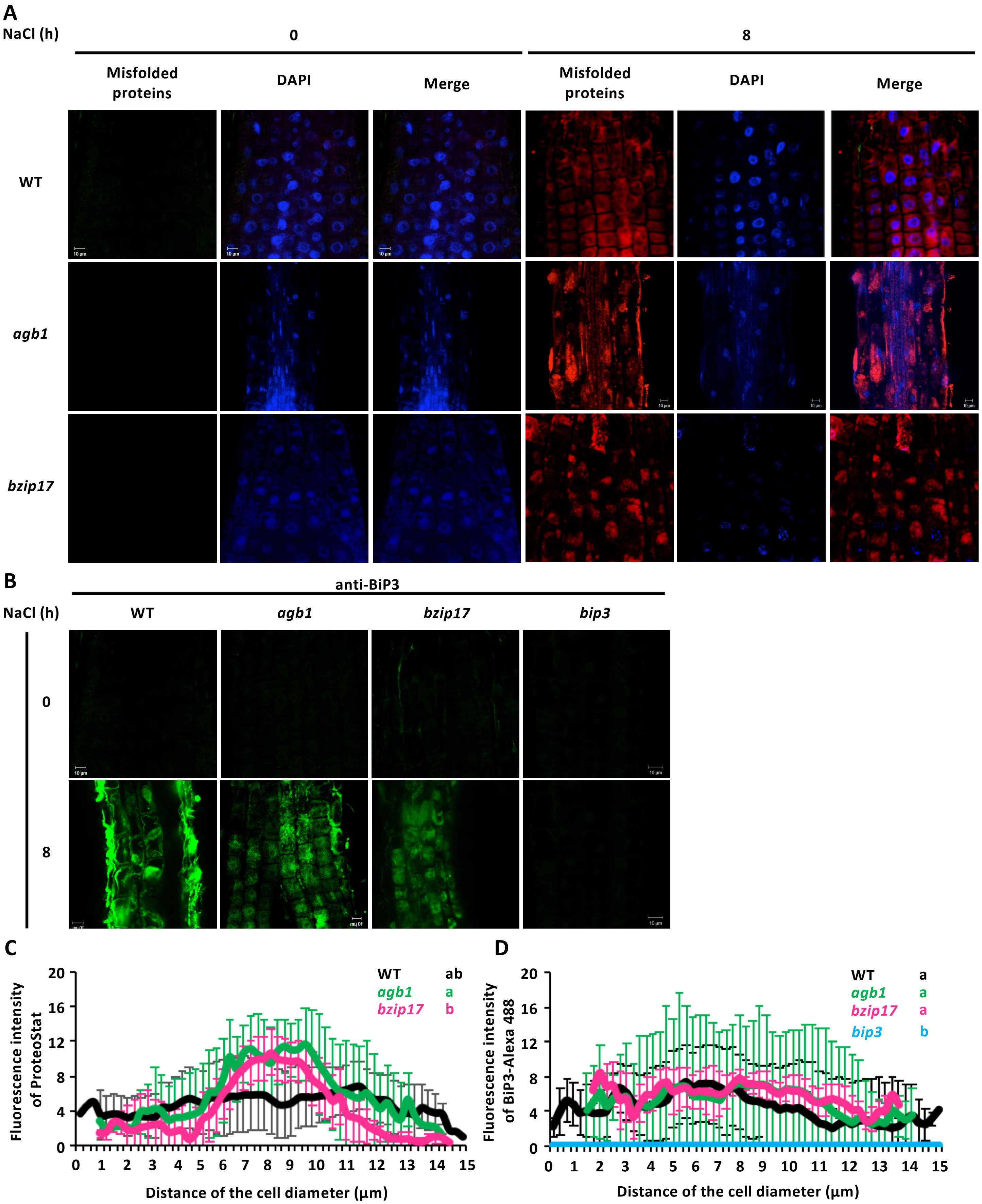

Supplemental Figure 9. Detection of misfolded proteins and ER chaperone BiP3 in salt-stressed roots.

Detection of misfolded proteins using high salinity treatment in meristemic zone of roots. Seven-day-old WT, *agb1*, *bzip17*, *bip3* were treated with 150 mM NaCl for 8 h. The misfolded proteins were detected by Aggresome dye ProteoStat (Red), the nuclei localizations were confirmed by DAPI staining (Blue), and merged images (Merge). (**B**) BiP3 localization detected by immunohistochemistry with anti-BiP3 antibodies (Green). Quantification analyses of aggresome staining (**A**) or BiP3 detection (**B**) to be line chart as (**C**) or (**D**) respectively.

Figure S10

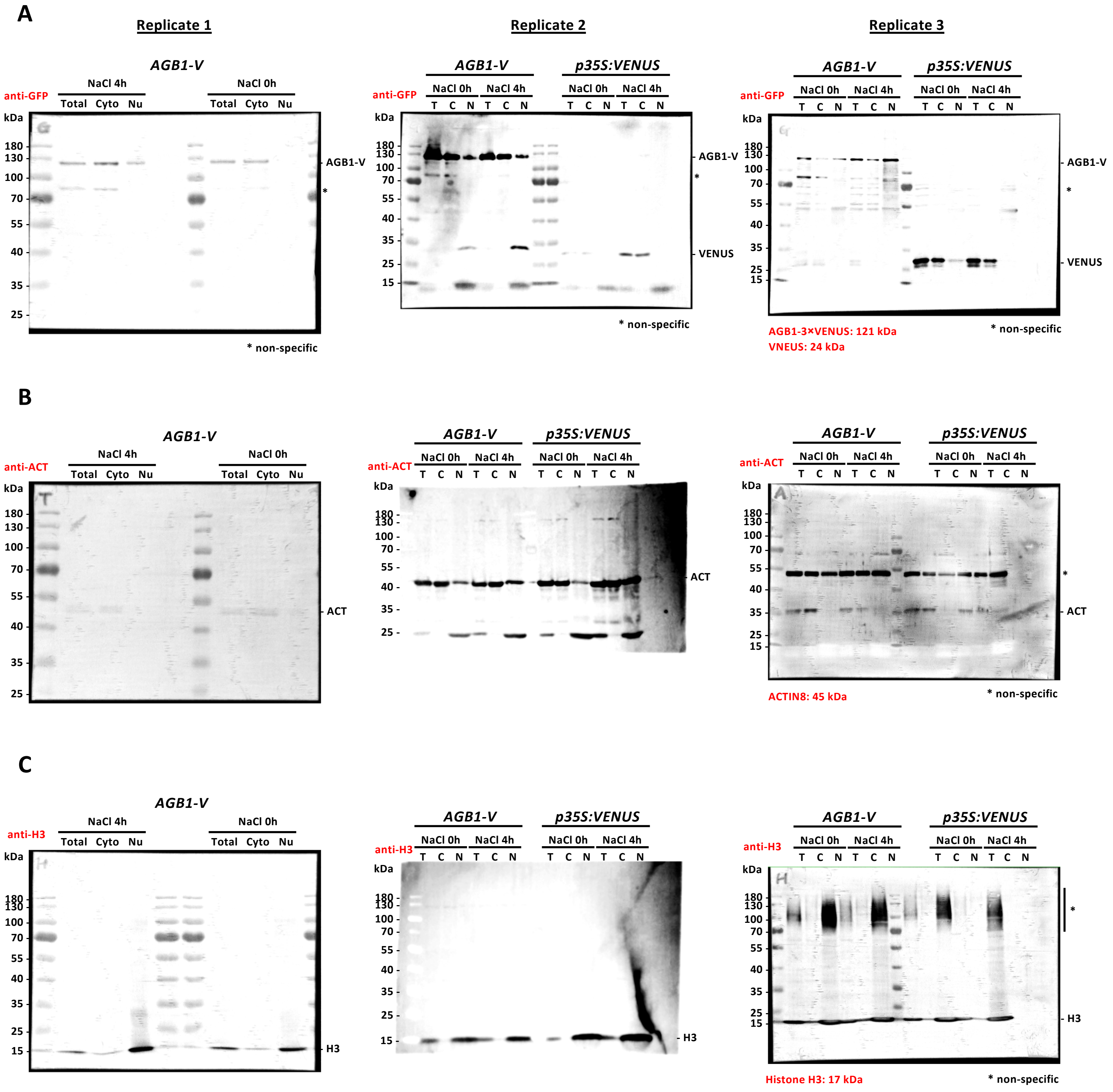

Supplemental Figure 10. Original images of immunoblot anti-GFP, anti-ACT8 and anti-Histone H3. The original images of immunoblots against GFP (for VENUS, **A**), ACTIN8 for cytosolic fractions (**B**) and Histone H3 (**C**) for nuclear fractions with replicates using 7-d *agb1-3 pAGB1:AGB1-Venus* (*AGB1-V*) and *p35S:VENUS* seedlings after 0- or 4-h of 150 mM NaCl treatment. Three replicates showed full length membranes, with membrane edges visible. The third replicate was used to prepare images showed in Fig. 2C. The size of anti-GFP blot was showed without crop further to cover both AGB1-V (121 kDa) and VENUS (24 kDa). For both anti-ACT8 and anti-H3 blots, the images were cropped from original one as displayed here.

Figure S11

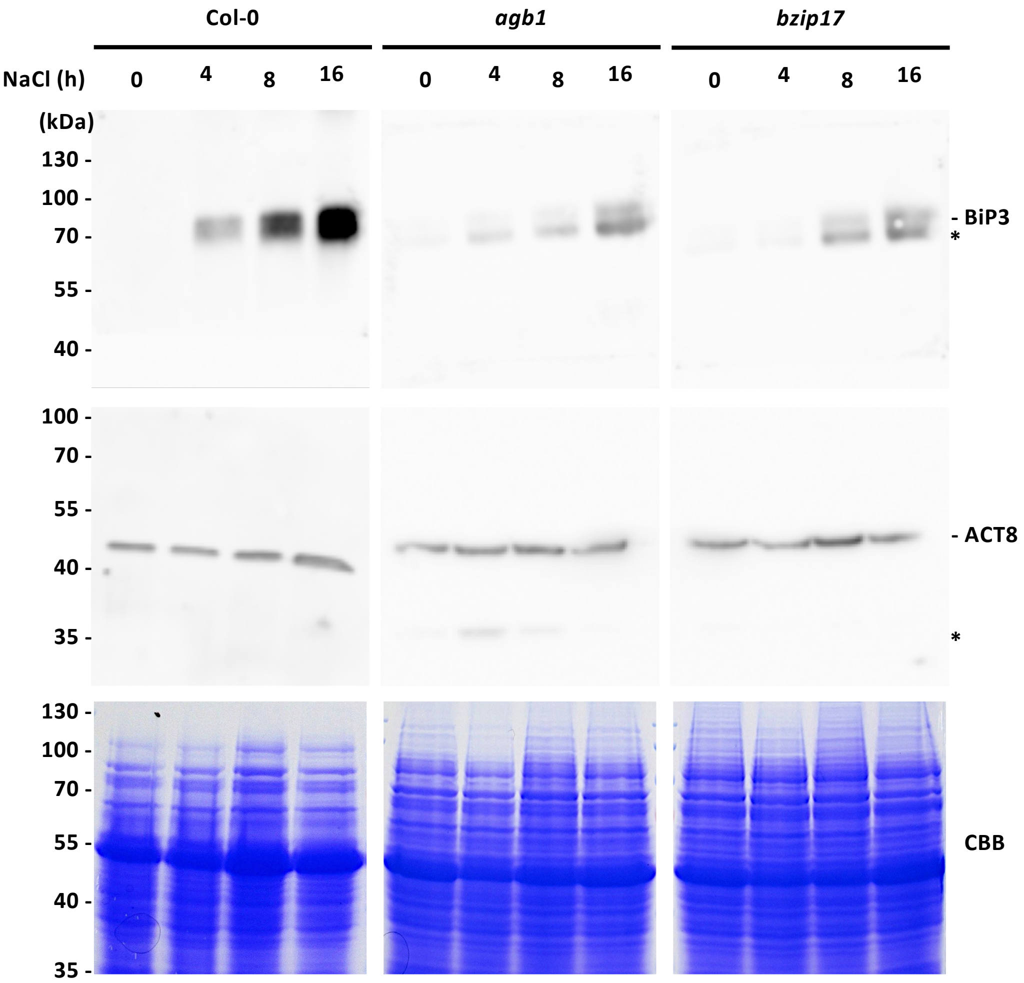

Supplemental Figure 11. Accumulation of BiP3 proteins by salt stress treatment.
Immunoblot analysis of BiP3 protein in WT, *agb1*, and *bzip17* seedlings in response to high salinity. (**A**) Total protein was extracted from 7-day-old seedlings treated with 150 mM NaCl for 4 h, and subjected to immunoblot analysis for BiP3 protein (top panel), ACT8 protein (middle panel), and CBB staining (bottom panel).

Figure S12

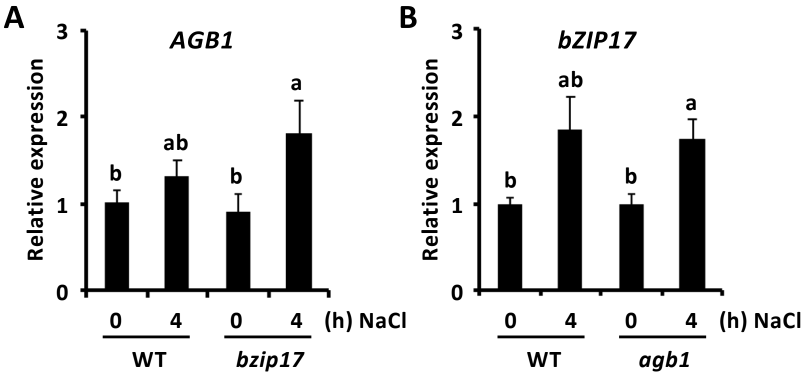

Supplemental Figure 12. *AGB1* and *bZIP17* expressions in *agb1* and *bzip17* mutants under high salinity.

(**A**) Quantitative RT-PCR analysis of 7-day-old WT, *agb1* or *bzip17* seedlings with 0 or 4 h of 150 mM NaCl treatment. The *AGB1* and *bZIP17* expressions were normalized to *ACTIN2* and average *AGB1* or *bZIP17* expression level under normal condition was set as 1. Data were mean ± SD of three biological replicates. Data with different letters represent significant differences [one-way ANOVA at $P < 0.05$].

Supplemental Figure S13

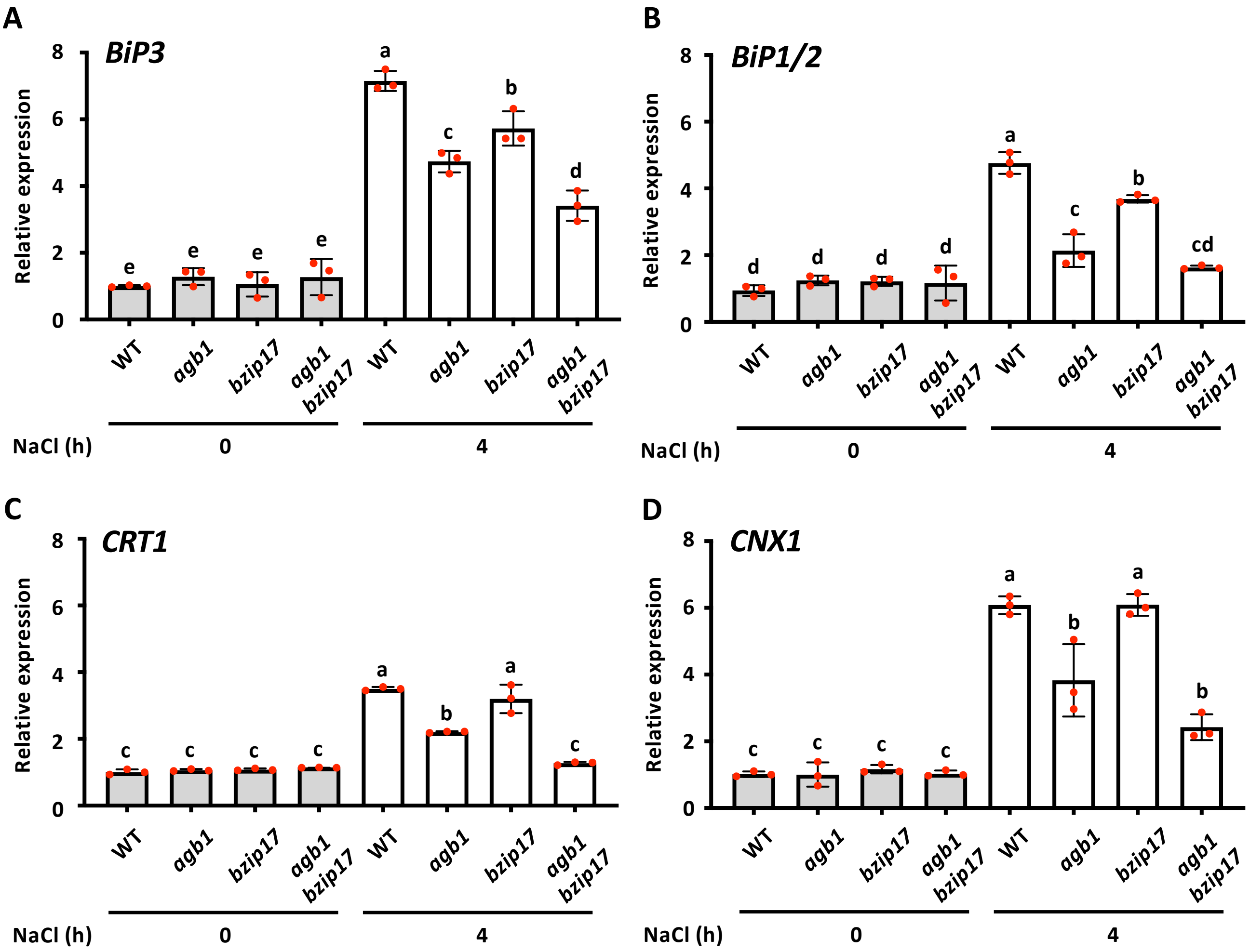

Supplemental Figure 13. Salt stress induction of UPR genes in the *agb1 bzip17* double mutant.
RT-qPCR analysis of the expression of the UPR genes (**A**) *BINDING PROTEIN 3* (*BiP3*), (**B**) *BINDING PROTEIN 1 and 2* (*BiP1/2*), (**C**) *CALRETICULIN 1* (*CRT1*) (**D**) CALNEXIN 1 (*CNX1*) in wild-type (WT), *agb1*, *bzip17* single mutants and *agb1 bzip17* double mutant plants in response to salt stress. Seedlings were grown on 1/2 MS agar plates for 7 days, and then transferred to 1/2 MS liquid medium containing 150 mM NaCl for the times indicated. The expression of the WT sample at 0 hour set to 1. Three technical replicates averaged data in the same run, and three biological replicates in separate runs were shown in mean ± SD. Data with different letters represent significant differences [one-way ANOVA at $P < 0.05$].

Figure S14

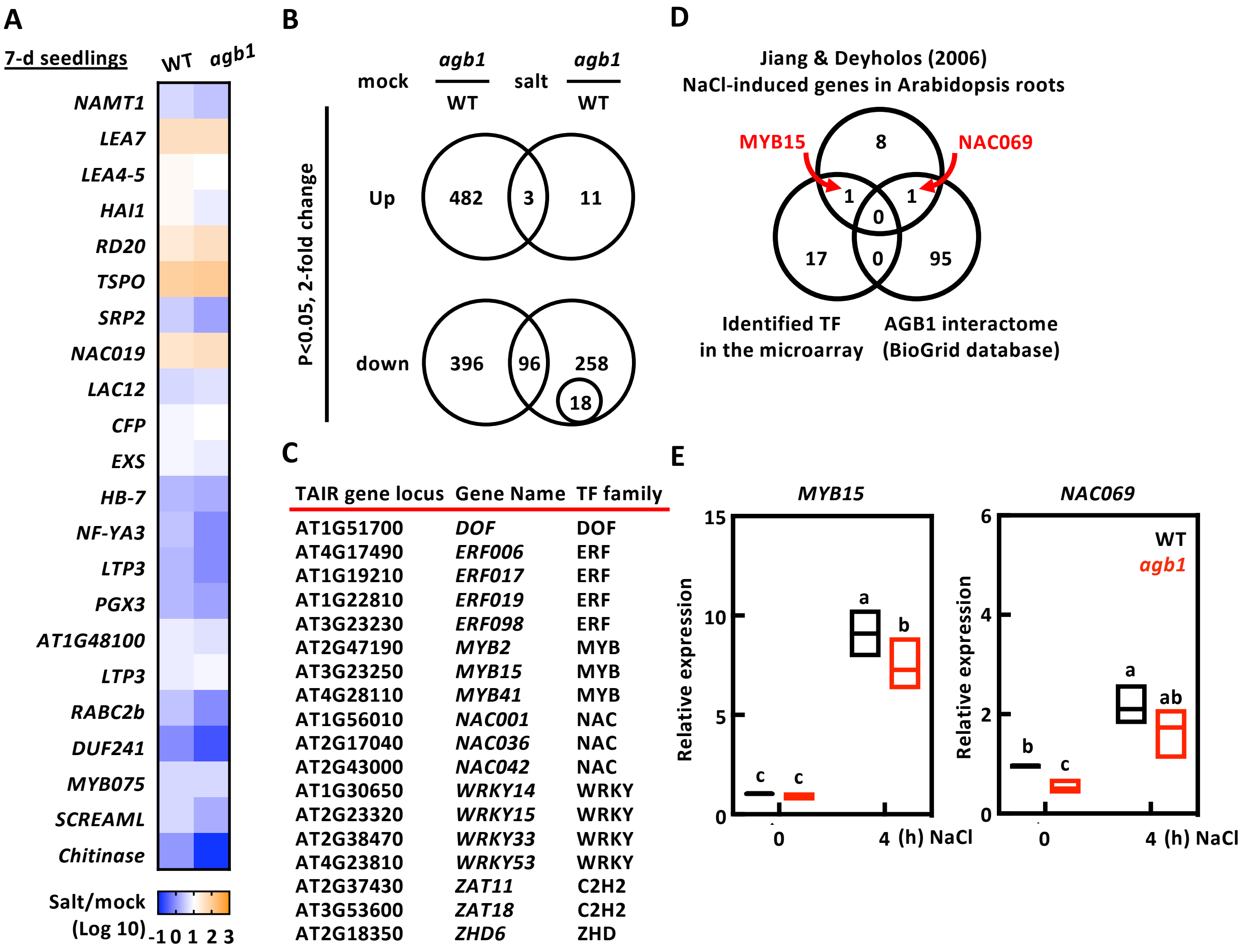

TAIR gene locus	Gene Name	TF family
AT1G51700	*DOF*	DOF
AT4G17490	*ERF006*	ERF
AT1G19210	*ERF017*	ERF
AT1G22810	*ERF019*	ERF
AT3G23230	*ERF098*	ERF
AT2G47190	*MYB2*	MYB
AT3G23250	*MYB15*	MYB
AT4G28110	*MYB41*	MYB
AT1G56010	*NAC001*	NAC
AT2G17040	*NAC036*	NAC
AT2G43000	*NAC042*	NAC
AT1G30650	*WRKY14*	WRKY
AT2G23320	*WRKY15*	WRKY
AT2G38470	*WRKY33*	WRKY
AT4G23810	*WRKY53*	WRKY
AT2G37430	*ZAT11*	C2H2
AT3G53600	*ZAT18*	C2H2
AT2G18350	*ZHD6*	ZHD

Supplemental Figure 14. Schematic representation of AGB1-mediated salinity response through bZIP17 signaling in Arabidopsis roots.

(**A**) Twenty two bZIP17-mediated salt stress responsive genes were selected from Liu *et al.*, (2007) and their expressions under salt stress in 7-day-old WT or *agb1-3* (*agb1*) mutant were shown as heatmap. Whole seedlings were collected after 0 (control) or 4 hours (salt) of 150 mM NaCl treatment for microarray analysis. The Log10 values indicated the means of the ratio of the representative transcripts under salt stress (4-h) comparing the mock condition in WT or *agb1* mutant from three independent replicates. (**B**) The AGB1-regulated salt responsive genes were identified by intersection of *agb1*/WT between control and salt condition. The up- or down-differential expression genes (DEG) were selected by two-fold differences (*P*<0.05 threshold for total genes in parentheses). (**C**) The salt stress responsive transcription factors from the down-DEG group of the *agb1*/WT under salt condition. (**D**) Venn diagram of selected ten genes from microarray and confirmed by RT-qPCR in Jiang & Deyholos (2006), identified 18 TFs with lower salt stress induction in *agb1* seedlings in our study (**C**), and 96 AGB1 interactors reported in the BioGrid database (https://thebiogrid.org). The overlap genes *MYB15* and *NAC069* were highlighted in red. (**E**) RT-qPCR analysis of 7-day-old WT (black) and *agb1* (red) seedlings with 0 or 4 h of 150 mM NaCl treatment. The *MYB15* (left) and *NAC069* (right) expressions were normalized to *ACTIN2* and average of each gene expression level under normal condition was set as 1. Data were mean ± SD of three biological replicates. Data with different letters represent significant differences [one-way ANOVA at P < 0.05].

Figure S15

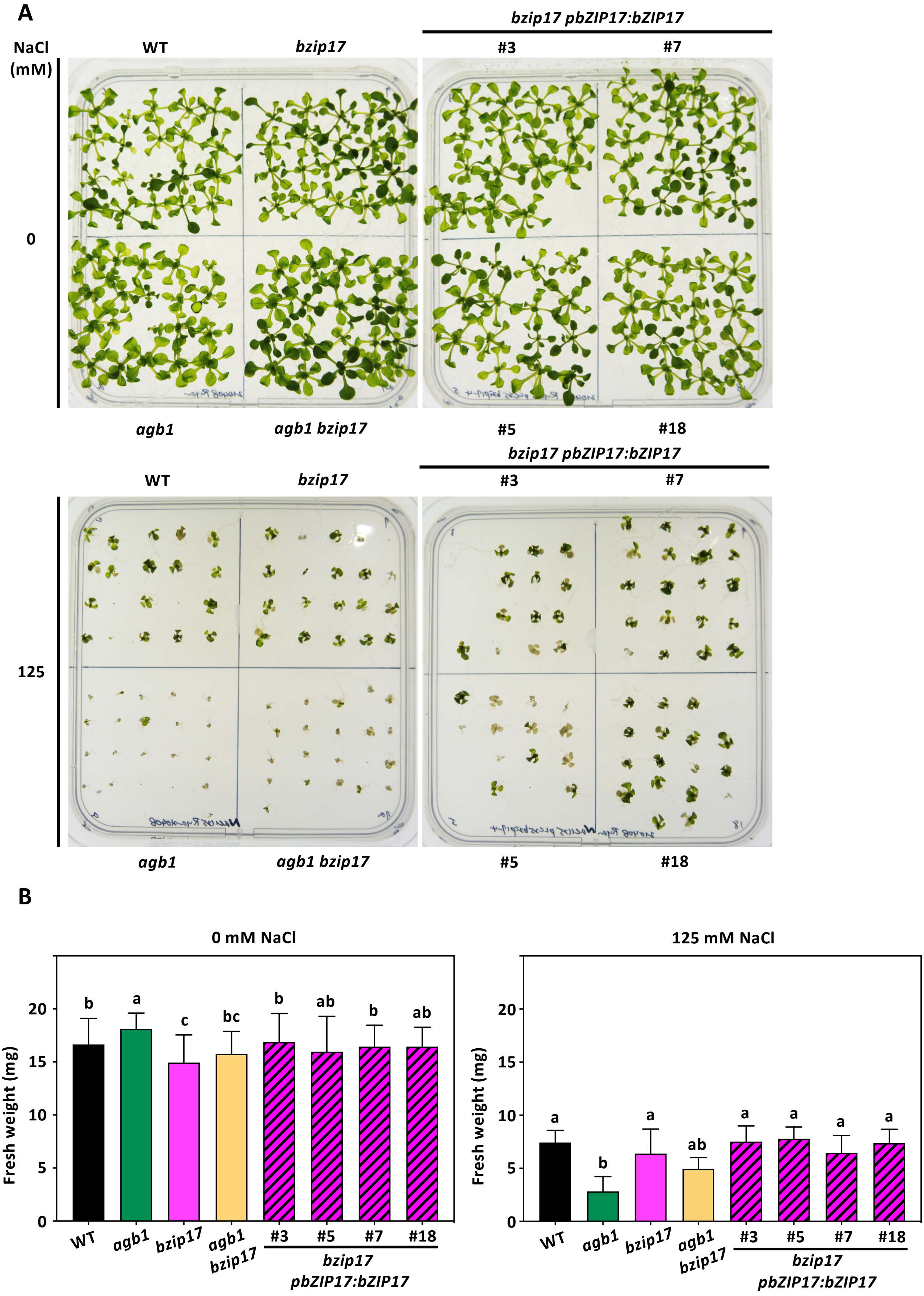

Supplemental Figure 15. Additional bZIP17 genomic DNA complementation lines are biological functioned for seedling growth.

Representative images (**A**) of 14-day-old WT, *agb1*, *bzip17*, *agb1 bzip17* mutants, and *bzip17* pbZIP17:bZIP17 complementation lines were grown on ½ MS containing NaCl with indicated concentration to induce salt stress. (**B**) Representative plots for fresh weight of 14-day-old seedlings grown on 0 (left) or 125 mM NaCl (right) were measured individually with three biological experiments (n=20) and shown as mean+SD. Data with different letters represent significant differences [one-way ANOVA at $P < 0.05$].

Figure S16

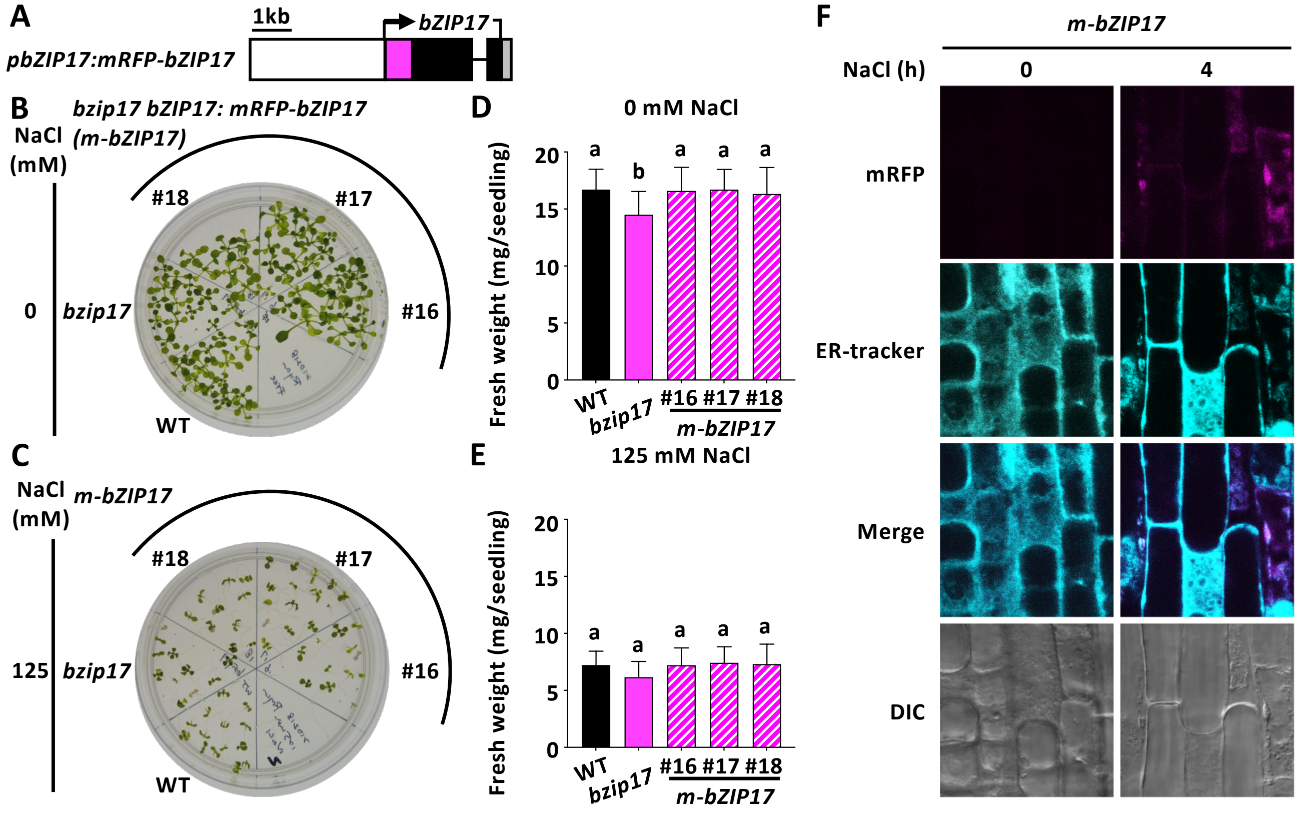

Supplemental Figure 16. Salinity tolerance and subcellular localization of transgenic mRFP-bZIP17 plants.

(**A**) The gene structure of the genomic *bZIP17* fragment with N-terminal fusion of mRFP reporter after ATG start codon. White box as the promoter, black boxes as two exons, black line as intron, gray box as 3'UTR and pink box as mRFP reporter. Representative images (**B**, **C**) of 14-day-old *WT*, *bzip17* mutants, and *bzip17 pbZIP17:mRFP-bZIP17* (m-bZIP17) complementation lines #16, #17, #18 were grown on ½ MS containing NaCl with indicated concentration to induce salt stress. (**D**, **E**) Representative chart for fresh weight of 14-day-old seedlings grown on 0 (up) or 125 mM NaCl (down) were measured individually with three biological experiments (n=15) and shown as mean+SD. Data with different letters represent significant differences [one-way ANOVA at $P < 0.05$]. (**F**) Subcellular localization of mRFP-bZIP17 in root of 7-day-old m-bZIP17 plants. Fluorescence of mRFP-bZIP17 (pink) and staining of the endoplasmic reticulum by ER-tracker in the treatment of 150 mM NaCl for 0 or 4 hours. Co-localization signals of mRFP-bZIP17 and staining dye were shown in the merge images as purple colors in the bottom panel. Differential interference contrast (DIC) images to show cellular structures. Scale bars equal to 10 µm.
